# Single cell transcriptomic analyses implicate an immunosuppressive tumor microenvironment in pancreatic cancer liver metastasis

**DOI:** 10.1038/s41467-023-40727-7

**Published:** 2023-08-23

**Authors:** Shu Zhang, Wen Fang, Siqi Zhou, Dongming Zhu, Ruidong Chen, Xin Gao, Zhuojin Li, Yao Fu, Yixuan Zhang, Fa Yang, Jing Zhao, Hao Wu, Pin Wang, Yonghua Shen, Shanshan Shen, Guifang Xu, Lei Wang, Chao Yan, Xiaoping Zou, Dijun Chen, Ying Lv

**Affiliations:** 1 Department of Gastroenterology, Nanjing Drum Tower Hospital, The Affiliated Hospital of Nanjing University Medical School, Nanjing, 210008 China; 2 Nanjing University Institute of Pancreatology, Nanjing, 210008 China; 3 State Key Laboratory of Pharmaceutical Biotechnology, School of Life Sciences, Nanjing University, Nanjing, 210023 China; 4 Department of Gastroenterology, Nanjing Drum Tower Hospital Clinical College of Jiangsu University, Nanjing, 210008 China; 5 Department of General Surgery and Pancreatic Disease Research Center, The First Affiliated Hospital of Soochow University, Suzhou, 215006 China; 6 Department of Pathology, Nanjing Drum Tower Hospital, The Affiliated Hospital of Nanjing University Medical School, Nanjing, 210008 China

**Keywords:** Cancer microenvironment, Metastasis, Pancreatic cancer

## Abstract

Pancreatic ductal adenocarcinoma (PDAC) is a highly metastatic disease refractory to all targeted and immune therapies. However, our understanding of PDAC microenvironment especially the metastatic microenvironment is very limited partly due to the inaccessibility to metastatic tumor tissues. Here, we present the single-cell transcriptomic landscape of synchronously resected PDAC primary tumors and matched liver metastases. We perform comparative analysis on both cellular composition and functional phenotype between primary and metastatic tumors. Tumor cells exhibit distinct transcriptomic profile in liver metastasis with clearly defined evolutionary routes from cancer cells in primary tumor. We also identify specific subtypes of stromal and immune cells critical to the formation of the pro-tumor microenvironment in metastatic lesions, including RGS5^+^ cancer-associated fibroblasts, CCL18^+^ lipid-associated macrophages, S100A8^+^ neutrophils and FOXP3^+^ regulatory T cells. Cellular interactome analysis further reveals that the lack of tumor-immune cell interaction in metastatic tissues contributes to the formation of the immunosuppressive microenvironment. Our study provides a comprehensive characterization of the transcriptional landscape of PDAC liver metastasis.

## Abstract (web summary)

The metastatic tumour microenvironment in pancreatic ductal adenocarcinoma (PDAC) remains to be explored. Here, the authors perform single cell RNA sequencing analysis for synchronously resected PDAC primary tumours and matched liver metastases and find differences in cellular composition.

## Introduction

Pancreatic ductal adenocarcinoma (PDAC) persists as one of the world’s most formidable and lethal malignancies, with a 5-year survival rate below 10%^1,2^. Often detected at a late stage, PDAC exhibits a staunch resistance to existing therapies, in part due to an exceedingly high incidence of distant metastasis (>80%) at initial diagnosis. The liver emerges as the predominant location for distant metastasis in PDAC cases. Presently, therapeutic approaches for PDAC patients with liver metastases remain markedly scarce^3^. Consequently, there is an urgent need to elucidate the molecular mechanisms that drive hepatic metastasis in PDAC, which could pave the way for the development of more efficacious treatments and ultimately enhance the survival rates for patients suffering from advanced stages of the disease.

PDAC is one of the least immune-infiltrated cancers, and is characterized by a complex tumor microenvironment (TME), defined by the interactions among multiple cell types, including malignant, immune, and stromal cells^4–6^. Tumor metastasis is a multistep process driven by both the intrinsic properties of tumor cells like mutational burdens, and the crosstalk between cancer cells and other cell types in the TME, including lymphoid cells, tumor-associated macrophages (TAM), cancer-associated fibroblasts (CAF), and components of the extracellular matrix (ECM)^7–10^. Understanding the interplay of various cell types in the TME is crucial to understanding tumor development, metastasis, and prognosis. Recent studies have reported mutational and transcriptional signatures in PDAC metastases through bulk genomic and transcriptomic sequencing;^11,12^ however, these datasets provide limited information on the molecular events driving the metastatic microenvironment in advanced PDAC.

Single-cell RNA sequencing (scRNA-seq) is a powerful tool that can delineate the gene expression pattern of each individual cell and decode the interactions among diverse cellular components in the TME^13–15^. Single-cell transcriptomic profiling of PDAC primary tumors has been systematically investigated, revealing that the immune and stromal landscapes in each patient are highly heterogeneous^4,5^, and that cytotoxic T cells with exhausted gene expression patterns might contribute to the immunosuppressive TME^16^. However, because metastatic PDAC are generally unresectable according to the NCCN Clinical Practice Guidelines, few studies have looked into the single-cell transcriptomic features of metastatic lesions. Two recent studies reported the differences in the TME of primary and metastatic PDAC lesions using limited number of unpaired biopsy tissues^17,18^. However, a comprehensive transcriptomic profiling of the TME in matched primary tumor and liver metastases of the same PDAC patient at single-cell resolution is still lacking. Emerging evidences have shown that synchronous surgeries of primary PDAC tumor and hepatic oligometastasis may achieve encouraging survival outcomes in highly selective PDAC cases^19^.

In this work, we comprehensively analyze the TME landscape of synchronously resected PDAC primary tumors and matched liver metastases. Using scRNA-seq, we characterize the differential cell population distribution and intercellular interactions between primary tumors and liver metastases. We not only uncover the distinct transcriptomic properties of tumor cells between primary and metastatic sites, but also identify specific subtypes of stromal and immune cells that might contribute to the formation of the immunosuppressive microenvironment in metastatic lesions. These results provide useful mechanistic information for the understanding of PDAC liver metastasis and the development of personalized therapies for metastatic PDAC patients.

## Results

### Single-cell transcriptomic atlas and cell typing in PDAC primary tumors and liver metastatic lesions

**Fig. 1:**
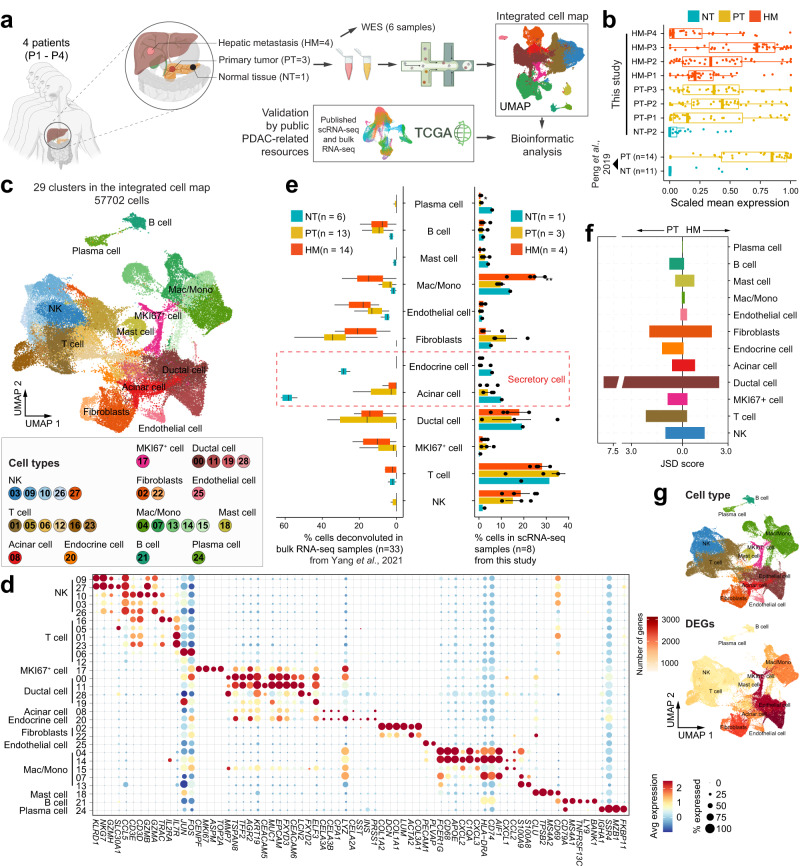
Single-cell transcriptomic analysis in primary and matched metastatic PDAC tissues. **a** Workflow of sample collection and data analysis in this study. **b** Boxplots showing the scaled mean expression of inflammation signatures (*n* = 42) in cells from different sample groups. The boxes indicate the median (horizontal line), second to third quartiles (box), and Tukey-style whiskers (beyond the box). The points indicate individual signatures. Similar patterns were observed in the PDAC scRNA-seq dataset from Peng et al.^5^. **c** Uniform Manifold Approximation and Projection (UMAP) plot displaying the integrated cell map, which consists of 29 cell clusters from 12 annotated cell types. Cells are colored by clusters. **d** Dot plot showing representative marker genes across cell clusters. Dot size is proportional to the fraction of cells expressing specific genes. Color intensity corresponds to the relative expression of specific genes. **e** Bar plot showing the cell type abundance for samples from different groups, as measured by scRNA-seq data in this study or deconvoluted bulk RNA-seq data from Yang et al.^12^. The error bar indicates standard error of the mean (s.e.m.). The *p* values are calculated using two-sided Wilcoxon rank-sum test. *p < 0.05; **p < 0.01. The boxes indicate the median (horizontal line), second to third quartiles (box), and Tukey-style whiskers (beyond the box). **f** Bar plot displaying the heterogenicity of cell types among different patients based on Jensen-Shannon divergence (JSD) score. **g** UMAP showing the distribution of major cell types (above) and the number of differentially expressed genes (DEGs) in each cell type.

To comprehensively understand the role of TME in PDAC metastasis, single-cell RNA sequencing (scRNA-seq, 10X Genomics) was carried out in eight fresh tissues collected from four PDAC patients (P1-P4), including three surgically resected primary pancreatic tumors (PT) and their respective paired hepatic metastases (HM), as well as one normal pancreatic tissue (NT) from one of the three patients (1 NT-PT-HM trio and 2 PT-HM pairs). Besides, one hepatic metastasis biopsy obtained by endoscopic ultrasound-guided fine needle aspiration (EUS-FNA) from a fourth patient was also included (Fig. 1a and Supplementary Table 1). The patients P1–P3 showed detectable *KRAS* mutation based on whole-exome sequencing (WES) and/or target gene sequencing. Patient P1 did not show KRAS mutation in WES data probably due to the low sequencing depth used (~100× coverage; Supplementary Fig. 1a). The *KRAS* mutation status of patient P4 was not determined because of no available samples. Notably, the expression levels of the inflammation-response genes were significantly higher in both PT and HM tissues than in the NT tissue, which was consistent with reanalyzed data from a previous study^5^, validating the tissue inflammation of the PT and HM samples (Fig. 1b). Following multiple quality control steps, we acquired single-cell transcriptomes in a total of 57,702 cells from all samples for downstream analysis. These cells were partitioned into 29 clusters of twelve main cell types, including ductal cells, T cells, natural killer (NK) cells, B cells, mast cells, plasma cells, endothelial cells, fibroblasts, myeloid cells, acinar cells, endocrine cells, and MKI67^+^ cycling ductal cells (Fig. 1c, Supplementary Fig. 1b and Supplementary Data 1), which were annotated by known markers (Fig. 1d and Supplementary Fig. 1c) and verified in an independent dataset consisted of primary pancreatic tumors^5^ (Supplementary Fig. 1d). Notably, the abundance of major cell types determined from the scRNA analysis was comparable to that estimated from immunofluorescence staining experiments (Supplementary Fig. 1e, f). To confirm the robustness and accuracy of data integration across different tissues, we performed data integration for PT and HM samples independently, and annotated cell types for each cell map separately. We observed that cell typing is quite consistent between the integrated cell map and the map of each tissue subset (Supplementary Fig. 1g). For example, two unique cell types, namely acinar cells and endocrine cells (collectively referred to as secretory cells), were identified in normal and cancerous pancreatic tissues but not hepatic metastasis tissues (Fig. 1e), further confirming the unbiased data integration and cell type annotation in our analysis.

Although nearly all cell clusters (except for secretory cells) were presented in all samples (Supplementary Fig. 1h, i), the proportion of each cell type was not evenly distributed across specimens (Fig. 1e and Supplementary Fig. 1i). We noticed that the proportion of ductal cells and fibroblasts was quite different in each patient (Fig. 1f), which is consistent with previous findings in other cancer types^13,15,20^. In contrast, the composition of immune cells was less heterogeneous among patients within the same tissue group. However, immune cells exhibited significant heterogeneity across different tissue types (Fig. 1e). Particularly, although the proportion of myeloid cells (mainly macrophages and monocytes) was similar across all patients within the same tissue group (Supplementary Fig. 1i), the fraction of myeloid cells in HM tissues was significantly higher than that in PT and NT tissues (Fig. 1e), raising an interesting question on the origin and function of these cells in the metastasis microenvironment that warrants future studies. Interestingly, the three cell types discussed above—ductal cells, fibroblasts and myeloid cells—that showed high heterogeneity among patients or tissue types, were also the cell types that harbored the most differentially expressed genes (DEGs) (Fig. 1g and Supplementary Fig. 1c), indicating highly dynamic transcriptional states of these cell types in different environment. Furthermore, we verified the above findings in the bulk RNA-seq data of a larger cohort of paired primary and metastatic PDAC samples^12^ using deconvolution analysis by CIBERSORTx^21^. We observed that the relative abundance of stromal cells and myeloid cells in our samples is consistent with the estimation from the published bulk RNA-seq data, while ductal cells and T cells show discordant patterns (Fig. 1e).

### Ductal cells show highly heterogeneous CNV patterns

**Fig. 2:**
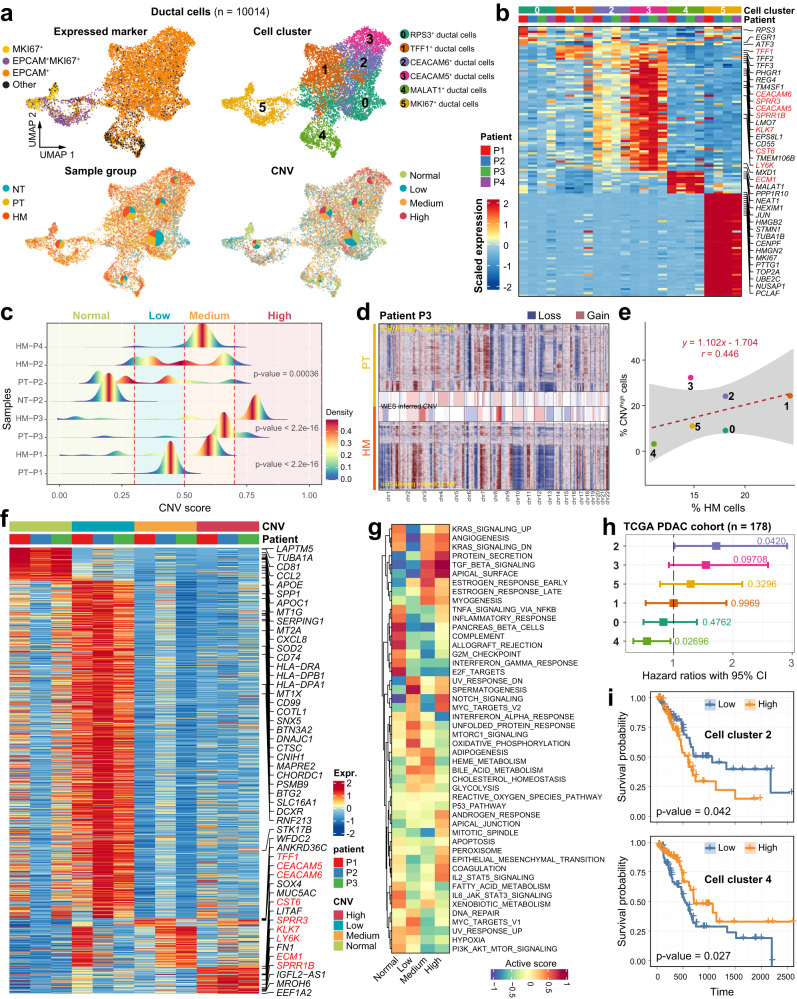
Transcriptional signatures and CNV heterogeneity of ductal cells. **a** UMAPs showing the distribution of ductal cells expressing the indicated maker genes (top left), ductal cell subtypes (*n* = 6; top right), the percentage of cells from different sample groups (bottom left) or at different CNV levels (bottom right) for each subcluster. **b** Heatmap showing the expression of marker genes in the six ductal cell subtypes. Selected marker genes are highlighted. **c** Ductal cells are grouped into different categories based on CNV score. Joyplots show the distribution of CNV score in different samples. Dashed lines in red indicate the threshold values. **d** CNV inferred by scRNA-seq and whole-exome sequencing (WES) data in patient P3. **e** The percentage of cells from HM samples is positively correlated with the proportion of cells with high level of CNVs. Fitted line and standard errors with 95% confidence intervals are shown. **f** Heatmap showing the scaled expression level of differentially expressed genes among cells with different CNV levels. Genes highlighted in red are also subtype-specific as shown in (**b**). **g** Heatmap showing functional pathways activated in cells with different CNV levels using GSVA analysis. **h** Association of relative cell abundance (estimated by CIBERSORTx) and patient survival using the TCGA PDAC cohort (*n* = 178) by COX regression analysis. The *p* value is calculated with two-sided log-rank test. **i** Kaplan–Meier curves of TCGA PDAC patients (*n* = 178) showing the survival rates grouped by the cell abundance in ductal cell clusters 2 and 4. The *p* value is calculated with two-sided log-rank test.

Since tumor cells are of ductal origin in PDAC, we first investigated the difference in tumor cells between PT and HM tissues by analyzing the gene expression profile in the ductal cell population. Of note, like ductal cells, we found that the majority of the MKI67^+^ cell cluster co-expressed the classical epithelial cell marker EPCAM (Fig. 2a), suggesting that the annotated MKI67^+^ cells are also of ductal origin. We therefore considered both EPCAM^+^ cells and MKI67^+^ cells as ductal cells and identified a total of 10,014 ductal cells from all 8 samples. The ductal cells were further divided into 6 subclusters through re-clustering analysis, including RPS3^+^, TFF1^+^, CEACAM6^+^, CEACAM5^+^, MALAT1^+^, and MKI67^+^ ductal cells, defined by a unique subset of highly expressed genes in each subcluster (Fig. 2a, b, Supplementary Fig. 2 and Supplementary Data 2). Cluster 5 ductal cells (MKI67^+^) were almost exclusively present in tumor tissues but not in NT control, suggesting that these are tumor cells of high proliferative capabilities (Fig. 2a and Supplementary Fig. 2c). In contrast, cluster 0 cells (RPS3^+^) were the major ductal cell population present in NT tissues, suggesting that these cells were the least malignant (termed “benign ductal cells”). The other four clusters were also predominantly present in tumor tissues (Fig. 2a and Supplementary Fig. 2c).

To further define the malignant state of the ductal cell subpopulations^22^, we inferred the single-cell CNV profile in ductal cells with inferCNV^23^ using myeloid cells as the reference (Fig. 2c). As expected, almost no CNV events were observed in the NT sample (Supplementary Fig. 3a). CNV in tumor samples exhibited high heterogeneity, with different degree of CNV accumulation across different patients and different tissue types (Fig. 2c). For instance, high CNV accumulation was enriched in certain chromosomes, such as chr7, chr8 and chr11, as validated by WES data in matched tumors (Fig. 2d and Supplementary Fig. 3b). We then divided all ductal cells into four groups based on the level of CNV: normal, low, medium and high (Fig. 2a, c and Supplementary Fig. 3c). Compared with NT and PT tissues, ductal cells from HM tissues exhibited remarkably higher CNV levels, indicative of more malignant phenotype (Fig. 2c, d and Supplementary Fig. 3a, b). This was also the case when the ductal cells in each HM sample were compared to the matched PT sample (Fig. 2c, d and Supplementary Fig. 3a, b). We also noticed that CNV patterns were highly subcluster-specific (Fig. 2a). In general, the percentage of CNV^high^ cells in each ductal cell subcluster was positively correlated with the percentage of HM cells in that cluster (Fig. 2e). We then compared the transcriptome among ductal cells with different levels of CNV (Fig. 2f). We observed that CNV^high^ ductal cells of HM tissues exhibited distinctive gene expression patterns from CNV^low^ ductal cells of PT tissues. Notably, a specific set of genes was almost exclusively expressed in the CNV^high^ ductal cells of HM tissues, including the kallikrein-related peptidase KLK7, carcinoembryonic antigen-related cell adhesion molecule CEACAM5, epidermal growth factor receptor kinase EPS8L1, extracellular matrix protein ECM1, lymphocyte antigen LY6K, cysteine proteinase inhibitor CST6, and small proline rich repeat proteins SPRR3 and SPRR1B (Fig. 2b, f, highlighted in red). Furthermore, pathway enrichment with gene set variation analysis (GSVA) demonstrated that TGF-β signaling, NOTCH signaling, MYC targets and epithelial-mesenchymal transition pathways were enriched in the CNV^high^ group (Fig. 2g).

To explore the clinical significance of the ductal cell subtypes identified in our study, we estimated the percentage of each ductal cell subcluster in patient samples from the TCGA PDAC cohort using CIBERSORTx^21^. Interestingly, the abundance of cluster 2 (CEACAM6^+^) and cluster 3 (CEACAM5^+^) ductal cells were both significantly correlated to poor overall survival (OS) (Fig. 2h, i and Supplementary Fig. 4a). In addition, the expression of these two marker genes as well as the abundance of these two clusters were higher in advanced-stage PDAC patients in the TCGA cohort (Supplementary Fig. 4b, c). In contrast, the abundance of cluster 4 (MALAT1^+^) was correlated with better OS, which is in line with the observation that this cluster highly expressed genes associated with “leukocyte mediated immunity”, “positive regulation of immune system process” and “positive regulation of lymphocyte activation” (Supplementary Fig. 4d).

### Developmental trajectory defines distinct ductal cell states and evolutionary dynamics from primary to metastatic PDAC

**Fig. 3:**
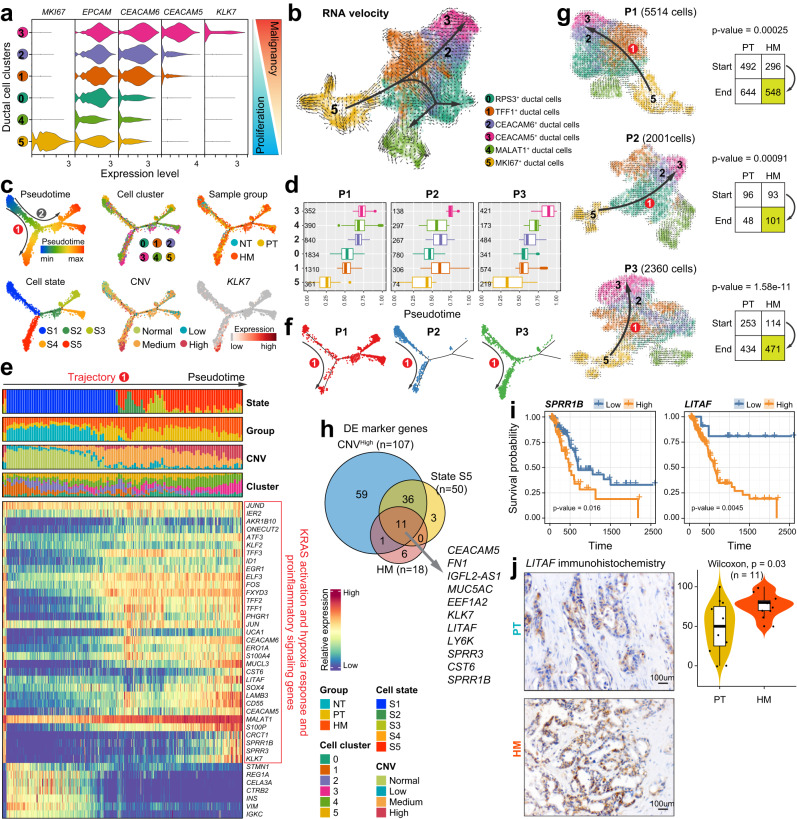
Pseudotime trajectory analysis reveals diverse ductal cell differentiation states. **a** Violin plots displaying the expression of representative genes associated with PDAC proliferation or malignancy across ductal cell subtypes. Color key from blue to red indicates relative marker genes expression pattern from proliferation to malignancy. **b** Unsupervised pseudotime trajectory of ductal cell subtypes by RNA velocity. Arrowhead direction represents the trend of cell pseudo-temporal differentiation. **c** Semisupervised pseudotime trajectory of ductal cell subtypes inferred by Monocle2. Trajectory is colored by pseudotime (top left), cell states (bottom left), cell subtypes (top middle), CNV levels (bottom middle), sample groups (top right) and the expression dynamics of a selected marker gene *KLK7* (bottom right). **d** Boxplot showing the latent time of ductal cell subtypes by RNA velocity in patients P1–P3. The number of cells in each category is indicated in the left of boxplot. The boxes showing the median (horizontal line), second to third quartiles (box), and Tukey-style whiskers (beyond the box). **e** Heatmap showing the scaled expression of differentially expressed genes across pseudotime trajectory in (**c**). Bar plots at the top of the heatmap are scale diagrams of different cell states, sample groups, CNV levels and cell subtypes during pseudotime differentiation trajectory. **f** Distribution of ductal cells along the Monocle2-estimated trajectories for patients P1–P3. **g** RNA velocity analysis of ductal cells for each patient (P1–P3). Arrowhead direction represents a conserved differentiation trajectory (from ductal cell subtype 5 to subtypes 2 and 3) among the patients. The number of ductal cells in PT and HM from the start subtype (cluster 5) and end subtypes (clusters 2 and 3) of the conserved trajectory (route 1) is shown on the right. The *p* value was calculated using the *χ*^2^ test. **h** Venn diagrams showing the overlap of the DEGs in high level CNV, cell state S5, and HM ductal cells. **i** Kaplan–Meier curves of patients in the TCGA PDAC cohorts (*n* = 178). The *p* value was calculated using the two-sided log-rank test. **j** Immunohistochemistry analysis (left) of LITAF expression in PT and HM groups. Violin plots (right) displaying immunohistochemical scores across the patients (*n* = 11). Scale bars, 100 μm. The boxes indicate the median (horizontal line), second to third quartiles (box), and Tukey-style whiskers (beyond the box). The error bar indicates standard error of the mean (s.e.m.). The *p* value is calculated with one-sided Wilcoxon rank-sum test.

As noted above, MKI67^+^ ductal cells (cluster 5) may function as a type of regenerative/proliferative tumor cell since they were exclusively found in tumor but not normal tissues, while CEACAM5^+^ ductal cells (cluster 3) may represent a class of malignant metastatic cells with high level of CNV that dominate the HM tissues (Fig. 2a). Across the six ductal cell subclusters, we observed an opposite trend of gene expression between proliferating markers (e.g., MKI67) and malignant metastatic markers (e.g., CEACAM5/6 and KLK7) (Fig. 3a), suggestive of orchestrated differentiation of tumor cells during metastasis. To delineate the evolutionary dynamics of pancreatic ductal cell lineages during PDAC progression and metastasis, we performed unsupervised cell trajectory analysis using both RNA velocity^24^ and Monocle2^25^. Both analyses revealed similar differentiation paths of ductal cells originating from stem-like/proliferating cells with two major branches (Fig. 3b, c), confirming the accuracy of trajectory prediction. Besides, the predicted pseudotime based on unsupervised RNA velocity analysis was similar in the three patients with paired tissues (P1–P3; Fig. 3d), indicating conserved differentiation trajectories among these patients. The pseudotime trajectory analysis based on Monocle2 defined five cell states (S1–S5) (Fig. 3c). Tissue-wise, ductal cells from the NT tissue were confined to S1, while ductal cells from PT and HM tissues were scattered among other states (S2–S5). Cell cluster-wise, MALAT1^+^ ductal cells (cluster 4) and MKI67^+^ ductal cells (cluster 5) dominate the S1 state, and showed up at the earliest stage during pseudotime in both PT and HM tissues, indicating their highly proliferative characteristics (Fig. 3c and Supplementary Fig. 5a, b). Of note, ductal cells in cluster 5 spanned over two distinct states with MKI67^+^EPCAM^−^ ductal cells enriched in state S1 and MKI67^+^EPCAM^+^ ductal cells enriched in state S3, indicating the existence of two different cell substates in cluster 5 (Supplementary Fig. 5c). Moreover, CEACAM5/6^+^ ductal cells (clusters 2/3) were the predominant subclusters in the S5 state, and only emerged at the latest stage in tumor tissues, demonstrating their highly malignant properties. Therefore, the ductal cells in clusters 2 and 3 are termed “pancreatic ductal adenocarcinoma cells”.

Two differentiation trajectories (routes 1 and 2; Fig. 3c) were identified, both rooted from S1. To recapitulate the transcriptomic characteristics of each trajectory, we overlayed the meta information about cell cluster, sample origin and CNV status with the predicted trajectory (Fig. 3d). The route 1 trajectory branched toward cell state S5 with high expression of oncogenes (CST6, LITAF, CRCT1, SPRR1B, SPRR3, and KLK7) (Fig. 3c, e and Supplementary Fig. 5d) and the ductal cell state transition along this trajectory was highly conserved among the patients (Fig. 3f). This trajectory showed an increased proportion of CNV^high^ ductal cells mostly derived from HM samples but a decrease in MKI67^+^ proliferative cells along the pseudotime. Therefore, route 1 resembled the development of metastatic cells along with the increased expression of genes regulated by KRAS activation (KLK7, ERO1A, SPRR3, TFF2, and AKR1B10) as well as hypoxia response and pro-inflammatory signaling genes (ATF3, JUN, EGR1, IER2, LITAF, FOS, LAMB3, ERO1A, S100A4, and KLF2). To rule out the potential influence of genetic variance on trajectory inference (Supplementary Fig. 3c), we performed RNA velocity analysis in each of the three patients with paired samples (P1–P3; Fig. 3g). We confirmed that the route 1 trajectory was independently validated in all the investigated patients. Interestingly, we observed a significant association (*χ*² test, *p* < 0.001) of state transition of ductal cells along trajectory 1 (from cluster 5 to clusters 2/3) and between the PT and HM groups (Fig. 3g), suggesting that trajectory 1 might recapitulate the state transition of ductal cells from PT to HM. In contrast, the route 2 trajectory was bifurcated into either S3 or S4, both states showing a mixture of cells with low-to-moderate CNV from PT and HM tissues (Fig. 3c). Interestingly, we found that genes associated with antigen processing and presentation of endogenous antigen were highly expressed in S3 and S4 (Supplementary Fig. 5e and Supplementary Data 3). We speculated that route 2 may reflect a differentiation progress related to antigen presentation.

Since S5 represented an end-point ductal cell state related to pancreatic cancer metastasis, we overlapped genes highly expressed in S5, genes that were overexpressed in ductal cells derived from HM tissues, and highly expressed marker genes in CNV^high^ cells. The Venn diagram showed eleven differentially expressed genes in common (Fig. 3h). This gene set may be used as a potential molecular signature for malignant metastatic cells. We then validated the expression pattern of these eleven genes using a published dataset^12^ with paired primary and metastatic PDAC samples and a PDAC scRNA-seq dataset^5^ (Supplementary Fig. 6a, b). Notably, the expression of LITAF and SPRR1B was much higher in metastatic than primary PDAC samples and the high expression of these two genes was significantly associated with poor prognosis in the TCGA dataset (Fig. 3i and Supplementary Fig. 6c, d). We also confirmed the upregulated protein expression of LITAF in HM tissues than in matched PT tissues using immunohistochemistry (IHC) in a cohort of PDAC patients (*n* = 11) from Nanjing Drum Tower Hospital (Fig. 3j). Besides, IHC analysis demonstrated elevated expression of LITAF in HM tissues than in paired PT tissues in another independent cohort of PDAC patients (*n* = 10) from the First Affiliated Hospital of Soochow University (Supplementary Fig. 6e). Moreover, we performed IHC staining of LITAF in a third cohort containing 46 PDAC tissues (PT) and adjacent normal pancreas (NT) collected at Nanjing Drum Tower Hospital. We found that LITAF expression was obviously higher in PT than in NT tissues (Supplementary Fig. 6f). Interestingly, we also noticed that LITAF expression was significantly increased in PDAC patients with lymph node metastasis compared with those without lymph node metastasis (Supplementary Fig. 6g). All these data suggest that LITAF expression is associated with the metastatic progression of PDAC.

### Cancer-associated fibroblasts subtyping and their contribution to the metastatic PDAC microenvironment

**Fig. 4:**
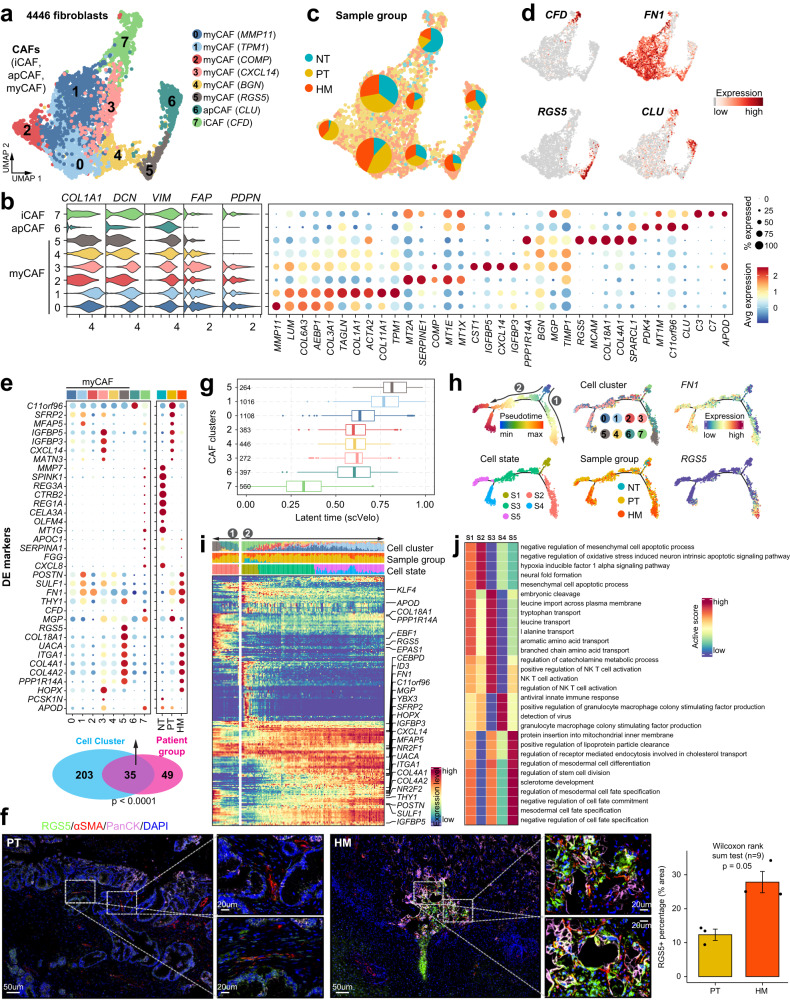
Transcriptional profiling of fibroblasts in the tumor microenvironment of primary and metastatic PDAC tissues. **a** UMAP showing the subtypes of cancer-associated fibroblasts (CAFs), including iCAF, apCAF and myCAF, colored by subclusters. **b** Violin plots (left) displaying the representative expression pattern across different subtypes of fibroblasts. Dot plot (right) showing the expression of the top five subtype-specific gene markers in each subtype. **c** Distribution of CAFs in different sample groups on the UMAP. Pie chart showing the proportion of three sample groups in each CAF subcluster. **d** Feature plots showing the expression of selected cluster-specific genes. Cells with the highest expression level are colored red. **e** Venn diagram (bottom) showing the overlap of DEGs between subclusters and sample groups of CAFs. The *p* value was calculated using the *χ*² test. Dot plot (top) showing the expression of these 35 DEGs across all stroma cell subclusters and sample groups. **f** Immunofluorescent staining showing co-localization of RGS5 (green), a-SMA (red), PanCK (purple), and DAPI (blue) in PT and HM samples. Scale bars, 50 μm (left) and 20 μm (right). The bar plots show the quantification results, *n* = 3 patients with paired PT and HM samples. The error bar indicates standard error of the mean (s.e.m.). The *p* value is calculated with one-sided Wilcoxon rank-sum test. **g** Boxplot showing the latent time of CAF subtypes by RNA velocity.The number of cells in each category is indicated in the left of boxplot (0: *n* = 1108; 1: *n* = 1016; 2: *n* = 383; 3: *n* = 272; 4: *n* = 446; 5: *n* = 264; 6: *n* = 397; 7: *n* = 560). The boxes showing the median (horizontal line), second to third quartiles (box), and Tukey-style whiskers (beyond the box). **h** Semisupervised pseudotime trajectory of CAF subtypes by Monocle2. Trajectory is colored by pseudotime (top left), cell states (bottom left), cell clusters (top middle), sample groups (bottom middle), and expression dynamics of two marker genes *FN1* and *RGS5* (right). **i** Heatmap showing the scaled expression of differentially expressed genes across pseudotime from **h**. Genes (listed to the right of the heatmap) are assigned to specific cell states based on their expression levels. Bar plots above the heatmap are scaled diagrams of different cell states, sample groups and cell clusters during pseudotime differentiation trajectory. **j** Heatmap showing the functional pathways enriched in five cell states (S1–S5) of CAFs by GSVA analysis.

Next, we analyzed the stromal compartment in the tumor microenvironment (Supplementary Fig. 7a and Supplementary Data 4), with an emphasis on fibroblasts (Fig. 4a). We found that all the fibroblast clusters showed a high expression of the well-defined panCAF markers including COL1A1, DCN, VIM, FAP, and PDPN^4^, indicating the identified fibroblasts are likely cancer-associated fibroblasts (CAFs) (Fig. 4b). CAFs are known to constitute the majority of the desmoplastic stroma and promote tumor growth and invasion through regulating extracellular matrix components in many cancer types including PDAC^26^, we thus focused on the expression signatures of CAFs in PDAC metastases. CAFs have been previously divided into inflammatory CAFs (iCAFs), myofibroblastic CAFs (myCAFs), and antigen-presenting CAFs (apCAFs) in pancreatic cancer^4^. We identified all three subtypes in our data based on known marker genes (Fig. 4a and Supplementary Fig. 7b). Clusters 0–5 were defined as myCAFs due to the expression of classical myofibroblastic markers such as ACTA2 (αSMA) and COL3A1; cluster 6 preferentially expressed CLU, C11orf96, and MT1M, representing an apCAF subtype; cluster 7 was identified as an iCAF subtype expressing the iCAF signature genes including CFD, C3, and C7. iCAFs and apCAFs are rare cell types and were mostly present in NT tissue in our data (Fig. 4c). Of the six myCAF clusters, cluster 5 (RGS5^+^ myCAF) was predominantly present in HM tissues, while the other five myCAF clusters showed similar distribution between PT and HM tissues (Fig. 4c and Supplementary Fig. 7c). All myCAF clusters, but not iCAFs and apCAFs, showed high expression of the fibronectin gene *FN1* (Fig. 4d), whose high expression is reported to be associated with aggressive pancreatic cancer^27^. We then compared the differentially expressed genes (DEG) among CAF clusters and the DEGs within CAFs among different tissues. We observed a significant overlap (*p* < 0.0001 by χ² test) between these two gene sets, suggesting these genes (*n* = 35) are the major contributors to transcriptional heterogeneity between CAFs of primary and metastatic tumors (Fig. 4e and Supplementary Fig. 8a). Notably, most of the upregulated DEGs in myCAFs, especially those related to cluster 5 RGS5^+^ myCAFs, including integrin (ITGA1), autoantigen (UACA), smooth muscle actin (RGS5), matrix Gla protein (MGP), collagen subunits (COL4A1, COL4A2, COL18A1), cell-surface biomarker (THY1) and fibronectin (FN1), were also highly expressed in HM tissues. The existence of RGS5^+^ myCAFs in PDAC was confirmed in a published scRNA-seq dataset^5^ (Supplementary Fig. 8b, c). Besides, the co-expression of αSMA and RGS5 was validated by multi-color immunofluorescence staining in both PT and HM tissues, confirming the existence of RGS5^+^ myCAFs in PDAC (Fig. 4f). Importantly, the accumulation of RGS5^+^ myCAFs surrounding ductal cells was remarkably higher in HM than in PT tissues, highlighting the potential biological significance of RGS5^+^ myCAFs in the metastatic microenvironment of PDAC.

To explore the development mechanisms and the potential role of these distinct CAF subpopulations in pancreatic cancer metastasis, we performed cell trajectory analysis to dissect their route of progression. Unsupervised trajectory analysis based on RNA velocity revealed that the CFD^+^ iCAF (cluster 7) was the earliest and most naïve CAF population, and the RGS5^+^ myCAF (cluster 5) was the most mature CAF population (Fig. 4g). Subsequently, we reordered the CAFs into pseudotime trajectories using Monocle2 by defining the CFD^+^ iCAF cluster as the starting point. This revealed five distinct cell states (S1–S5) and two major trajectory routes (routes 1 and 2; Fig. 4h). Interestingly, we noticed that both trajectories differentiated through intermediate CAFs derived from PT tumors and branched toward cell states enriched for myCAFs derived from HM tumors, suggesting both differentiation trajectories are related to pancreatic cancer metastasis (Fig. 4h). Route 1 consisted of states S1 and S2, where CAFs transitioned from the iCAF subtype to an intermediate state of apCAFs then to the RGS5^+^ myCAFs. This trajectory showed increased expression of the pericyte marker RGS5 and hypoxia-inducible factor EPAS1 (Fig. 4i) and enriched pathways related to mesenchymal cell apoptosis and hypoxia response (Fig. 4j). In contrast, CAFs in route 2 developed from iCAFs to myCAFs of distinct states which then bifurcated into either S4 or S5 states expressing high FN1 (Fig. 4h, i and Supplementary Fig. 9). Different from route 1, functional enrichment of gene markers in states from route 2 was highlighted by pathways related to granulocyte macrophage colony stimulating factor production (for S4) and mesodermal cell fate specification (for S5) (Fig. 4j). Taken together, our analysis reveals the accumulation of specific myCAF subclusters during PDAC metastatic progression.

### Lipid-associated macrophages (LAMs) play an important role in PDAC liver metastasis

**Fig. 5:**
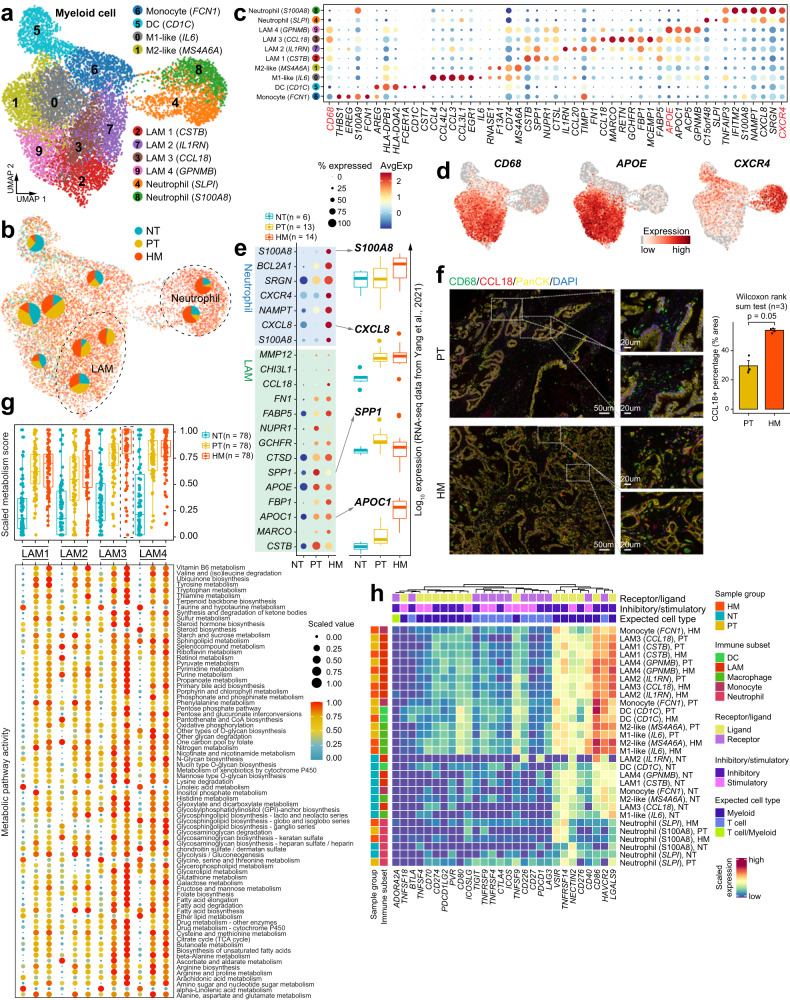
Transcriptional landscape of myeloid cells in the tumor microenvironment of primary tumor and liver metastasis tissues. **a** UMAP showing the subtypes of myeloid cells, colored by subtypes. **b** Distribution of myeloid cells in different sample groups on the UMAP. Pie chart showing the proportion of three sample groups in each cell subcluster. **c** Dot plot illustrating the average expression and frequency of representative marker genes in each myeloid cell subcluster. **d** Feature plots showing the expression of selected cluster-specific genes. Cells with the highest expression level are colored red. **e** Dot plot illustrating DEGs in neutrophils and Lipid-associated macrophages (LAMs) from three sample groups (left). Boxplots showing the expression patterns of *S100A8*, *CXCL8*, SPP*1*, and *APOC1* using the bulk RNA-seq dataset from Yang et al.^12^. The number of samples in each group is in the legend. The boxes showing the median (horizontal line), second to third quartiles (box), and Tukey-style whiskers (beyond the box). **f** Immunofluorescent staining showing co-localization of CD68 (green), CCL18 (red), PanCK (yellow), and DAPI (blue) in PT and HM samples. Scale bars, 50 μm (left) and 20 μm (right). The bar plots show the quantification results, *n* = 3 patients with paired PT and HM samples. The error bar indicates standard error of the mean (s.e.m.). The *p* value is calculated with one-sided Wilcoxon rank-sum test. **g** Boxplot (top) showing the metabolic score of metabolic pathways in four LAM subclusters (LAM1-LAM4). The points indicate individual pathways (*n* = 76). Dot plot (bottom) showing the metabolic activity analysis of all LAM subclusters by scMetabolism. The circle size and color darkness both represent the scaled metabolic score. The number of pathways in each category is indicated below the boxplot. The boxes showing the median (horizontal line), second to third quartiles (box), and Tukey-style whiskers (beyond the box). **h** Heatmap showing the scaled expression levels of a series of immune checkpoint genes in myeloid cell subtypes. Subtypes are grouped by sample source and myeloid cell type annotations (DC, LAM, macrophage, monocyte and neutrophil). Genes are grouped as receptor or ligand, inhibitory or stimulatory status and expected major lineage cell types known to express the gene (lymphocyte and myeloid).

Because the fraction of macrophage/monocytes in HM tissues was significantly higher than that in PT tissues (Fig. 1e), we investigated the composition and gene expression of myeloid-derived cells in PDAC liver metastases. Re-clustering of all 10062 myeloid cells revealed ten cell populations with varying frequencies in different tissues (Fig. 5a–c, Supplementary Fig. 10a and Supplementary Data 5). The monocyte population (cluster 6) was characterized by high expression of FCN1, S100A9, EREG, and THBS1 (Fig. 5c). We also identified a cluster of classical dendritic cells (DCs) expressing CD1C, CST7, and FCER1A, which most likely originated from normal pancreatic tissues (Fig. 5b). For the macrophage population (CD68^+^ cells, Fig. 5c, d), in addition to the conventional ‘M1-like’ (cluster 0) and ‘M2-like’ (cluster 1) cells, we discovered four macrophage clusters (clusters 2/3/7/9) that can all be classified as the recently discovered lipid-associated macrophages (LAMs)^28^. The four LAM clusters (LAM1-4) shared similar transcriptomic characteristics including high expression of the macrophage marker CD68 and lipid metabolism genes such as APOE, APOC1, and FABP5 (Fig. 5c, d). LAM1 and LAM2 both had high expression of SPP1, which has been reported to be secreted by TAMs to promote cancer progression^29^; while LAM3 and LAM4 showed high expression of CCL18 which has immune-suppressive and tumor-promoting functions^30^ (Fig. 5c). Moreover, we observed a substantially increased proportion of LAM1/2/3 cells in tumor tissues, of which the CCL18^+^ LAM3 cells were predominantly present in HM tissues (Fig. 5b). A closer look at the gene expression prolife of LAM3 among different tissue types showed the higher expression of lipid metabolism-associated genes like APOC1, APOE, and FABP5 and immune-related genes like CCL18 in HM than in PT and NT tissues, which was validated in a published dataset by Yang et al.^12^ (Fig. 5e, upper part; Supplementary Fig. 10b, c). The existence of CCL18^+^ macrophages was also verified in a published dataset by Peng et al. ^5^ (Supplementary Fig. 10d, e). Moreover, multi-color immunofluorescence staining confirmed the remarkably enhanced infiltration of CCL18^+^ macrophages (CD68^+^) in HM than PT tissues (Fig. 5f).

Gene ontology (GO) analysis revealed the enrichment of certain pathways in LAM cells such as regulation of lipid localization, fatty acid transport, triglyceride metabolism, inflammatory response, and regulation of immune system process, highlighting LAM as an important source of lipid metabolism and immunoregulatory molecules (Supplementary Fig. 10f and Supplementary Data 6). To understand the metabolic landscape of LAMs in PDAC metastasis, we utilized scMetabolism^31^ to systematically quantify the metabolic activities using our scRNA-seq data. We computed the metabolic pathway activity score for all 76 metabolic pathways annotated in scMetabolism, and found the metabolic score of LAMs in HM tissues was significantly higher than that in PT and NT tissues (Supplementary Fig. 10g). Surprisingly, the CCL18^+^ LAM3 cells enriched in HM tissues exhibited the highest metabolic activities among all LAMs, demonstrating that they were exceedingly vital and energetic at metastatic sites (Fig. 5g). Additionally, unsupervised clustering of all the metabolism-related genes within LAM3 cells identified more than 50 metabolic pathways that were markedly upregulated in HM tissues (Supplementary Fig. 11a). Among them, numerous lipid metabolism-associated pathways including glycerolipid metabolism, glycerophospholipid metabolism, fatty acid elongation and fatty acid degradation were highly enriched in HM-infiltrated LAM3 cells. We further investigated into the differentially expressed metabolic genes, and found that a large number of these genes, such as PLD3 (related to glycerophospholipid metabolism), LDHB (related to propanoate metabolism) and GSTO1 (an enzyme involved in metabolism of xenobiotics by cytochrome P450), were remarkably upregulated in HM-associated LAM3 (Supplementary Fig. 11a, b).

Neutrophils, which have been reported as an important component of the metastatic tumor microenvironment^32^, were prominently enriched in HM tissues (Fig. 5a, b and Supplementary Fig. 11c). We identified two populations of neutrophils: one (cluster 4, SLPI^+^ neutrophils) highly expressing SLPI, C15orf48, TNFAIP3, and the other (cluster 8, S100A8^+^ neutrophils) expressing IFITM2, S100A8, NAMPT, CXCR4, SRGN, and CXCL8 (Fig. 5a–c). Of note, the expression of most S100A8^+^ neutrophil markers were substantially elevated in HM tissues compared with PT and NT tissues, and S100A8 was almost exclusively expressed in HM tissues (Fig. 5e, bottom part). Consistent with our scRNA-seq data, the published dataset by Yang et al.^12^ also confirmed the high expression of most cluster 8 marker genes in metastatic PDAC, including S100A8, SGRN, NAMPT, CXCL8, and IFITM2 (Fig. 5e, right; Supplementary Fig. 11d). We further confirmed the increased infiltration of S100A8^+^ neutrophils in HM tissues by immunofluorescence staining (Supplementary Fig. 11e), indicating that the infiltration of S100A8^+^ neutrophils is closely linked to the metastatic progression of PDAC.

Myeloid cells have been reported as an important source of immune checkpoints in tumors^33^, we therefore analyzed the expression of immune checkpoint genes within the myeloid compartment in our samples. In general, the expression of immune checkpoint genes and their ligands showed distinctive patterns between myeloid cell subsets (Fig. 5h). Interestingly, we observed marked upregulation of a series of immune checkpoint genes including VISR, TNFRSF14, NECTIN2, CD40, CD86, CD276, HAVCR2, and LGALS9 in most tumor-infiltrating myeloid cells (Fig. 5h). We closely looked at the differentially expressed checkpoint genes in LAM3 cells; among them CD40, CD86, CD276, HAVCR2, and LGALS9 were highly expressed in HM tissues (Supplementary Fig. 12a). Most of these genes were specific for myeloid cells and regarded as immunosuppressive molecules (Supplementary Fig. 12b). Subsequent survival analysis demonstrated that high expression of CD276, CD40, and LGALS9 was significantly associated with poor prognosis in the TCGA PDAC dataset (Supplementary Fig. 12c). Collectively, these data suggest a crucial function of CCL18^+^ LAM3 cells in PDAC microenvironment especially in the metastatic microenvironment.

### Lymphoid reprogramming towards a metastatic microenvironment in PDAC

**Fig. 6:**
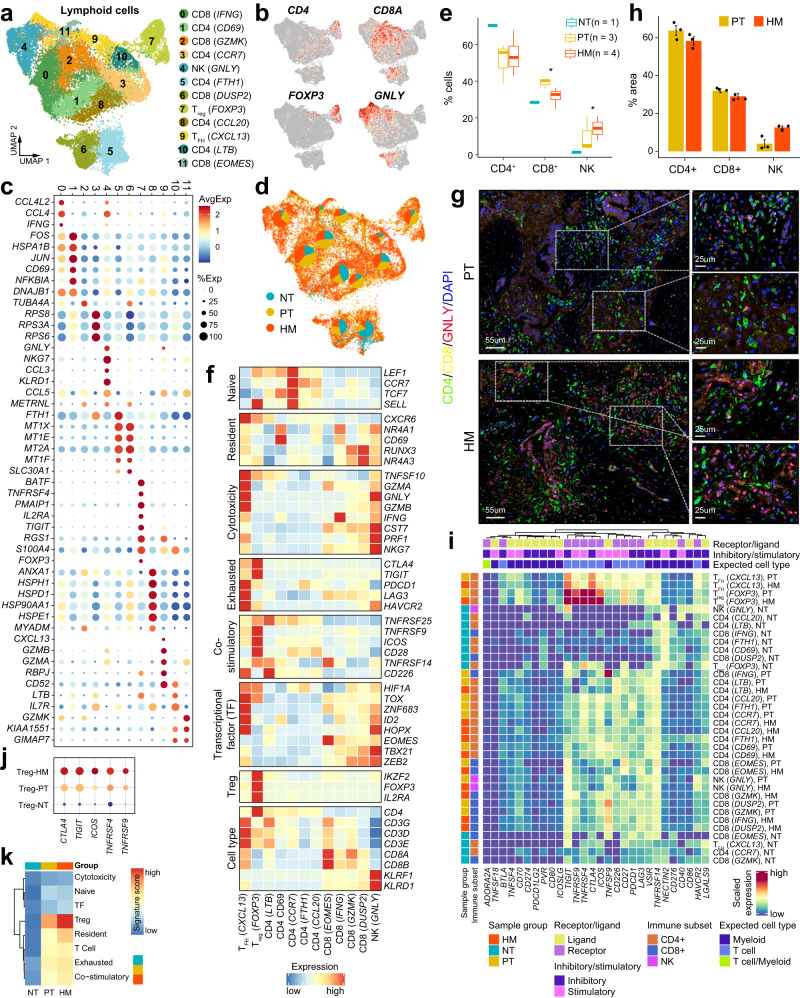
Transcriptional landscape of lymphoid cells in the tumor microenvironment of primary tumor and liver metastasis tissues. **a** UMAP projection of subclustered lymphoid cells, labeled in different colors. **b** Feature plots showing the expression of selected cluster-specific genes. Cells with the highest expression level are colored red. **c** Dot plot illustrating the average expression and frequency of representative marker genes in the subclusters of lymphoid cells. **d** Distribution of lymphoid cells in different sample groups on the UMAP. Pie chart showing the proportion of three sample groups in each cell subcluster. **e** Boxplot indicating the proportion CD4^+^ T, CD8^+^ T and NK cells in three sample groups (NT; *n* = 1, PT; *n* = 3, HM; *n* = 4). The boxes showing the median (horizontal line), second to third quartiles (box), and Tukey-style whiskers (beyond the box). The *p* value is calculated using one-sided Wilcoxon rank-sum test. *p < 0.05. **f** Heatmap indicating the expression of selected gene sets, including naive, resident, cytotoxicity, exhausted, co-stimulatory, transcriptional factors (TF), and cell type, in each lymphoid cell subcluster. **g** Immunofluorescent staining showing co-localization of *CD4* (green), *CD8* (yellow), *GNLY* (red), and DAPI (blue) in PT and HM samples (*n* = 3). Scale bars, 55 μm (left) and 25 μm (right). **h** The bar plots show the quantification results from **g**, *n* = 3 patients with paired PT and HM samples. The error bar indicates standard error of the mean (s.e.m.). **i** Heatmap showing the scaled expression levels of a series of immune checkpoint genes in subtypes of lymphoid cells. Subtypes are grouped by sample source and lymphocyte cell type annotations (CD4^+^ T, CD8^+^ T, and NK cell). Genes are grouped by receptor or ligand, inhibitory or stimulatory status and expected major lineage cell types known to express the gene (lymphocyte and myeloid). **j** Dot plot illustrating the expression levels of five checkpoint genes (*CTLA4*, *TIGIT*, *ICOS*, *TNFRSF4*, and *TNFRSF9*) in regulatory T cells from three sample groups. **k** Heatmap indicating the expression of selected gene sets, including cytotoxicity, naive, transcriptional factors (TF), resident, cell type, exhausted, and co-stimulatory, in regulatory T cells from three sample groups.

We next characterized the transcriptional properties of the lymphoid cell population. Unsupervised clustering of lymphoid cells resulted in twelve subclusters, including seven CD4^+^ T cell clusters (CD4 CD69^+^, CD4 CCR7^+^, CD4 FTH1^+^, CD4 CCL20^+^, CD4 LTB^+^, T_reg_ FOXP3^+^, and T_FH_ CXCL13^+^), four CD8^+^ T cell clusters (CD8 IFNG^+^, CD8 GZMK^+^, CD8 DUSP2^+^, and CD8 EOMES^+^) and one NK cell cluster (NK GNLY^+^) (Fig. 6a–c and Supplementary Data 7). All these subpopulations were shared among NT, PT, and HM tissues (Fig. 6d). Notably, NK cells, which displayed high expression levels of GNLY, NKG7, CCL3, and KLRD1 (Fig. 6c), were enriched in tumor tissues, especially in HM tissues (Fig. 6d, e). In addition, NK cells showed remarkable upregulation of cytotoxic gene expression (Fig. 6f). Immunofluorescence staining also indicated an increase of GNLY^+^ NK cells in HM tissues compared with PT tissues (Fig. 6g, h), implying an important role of NK cells in the metastatic growth of PDAC. Similarly, FOXP3^+^ T_reg_ cells were heavily enriched in tumors, both in PT and HM tissues (Fig. 6d), demonstrating both “co-stimulatory” and “exhausted” signatures (Fig. 6f). The T_reg_ cells also expressed remarkably high levels of immune checkpoint genes including CTLA4, TIGIT, ICOS, TNFRSF4, and TNFRSF9 (Fig. 6i). Notably, the inhibitory molecules CTLA4 and TIGIT were predominantly expressed in T_reg_ and T_FH_ cells, but rarely expressed in other T cell clusters (Fig. 6i). More importantly, we observed significantly higher expression of all the five checkpoint genes within T_reg_ cells including CTLA4, TIGIT, ICOS, TNFRSF4, and TNFRSF9 in HM tissues than PT tissues (Fig. 6j). Besides, HM-enriched T_reg_ cells displayed more prominent “Treg” and “resident” features, suggesting a potential role of FOXP3^+^ T_reg_ cells in metastatic PDAC (Fig. 6k). The CXCL13^+^ T_FH_ cells were also enriched in tumors with high expression of cytotoxic genes like GZMA, GZMB, TNFSF10, and GNLY (Fig. 6f). However, the proportion of T_FH_ cells was lower in HM tissues than in PT tissues (Fig. 6d). Notably, the CXCL13^+^ T_FH_ cells showed high expression of immune checkpoints proteins including PDCD1 (PD-1), LAG3, and HAVCR2 known to enhance T cell cytotoxicity (Fig. 6f). Together, these data suggest that multiple immune checkpoint genes were expressed in the T_reg_ and T_FH_ cell populations. In particular, some checkpoint genes were extremely upregulated in T_reg_ cells within the metastatic microenvironment (HM tissues).

For CD8^+^ T cells, the four defined subpopulations were all abundantly present in PDAC tumors, and similarly distributed between HM and PT tissues (Fig. 6a, d). Nearly all the CD8^+^ T cells in tumor tissues exhibited low levels of “co-stimulatory” and “exhausted” gene signatures, but high levels of “resident” and “cytotoxicity” gene expression (Fig. 6f). For example, the EOMES^+^ CD8 T cells, the proportion of which was slightly higher in HM tissues than in PT tissues, displayed high expression of EOMES, GZMK, and TNFRSF14, but reduced expression of exhausted genes including CTLA4 and TIGIT (Fig. 6f).

### Reprogrammed interactome landscape across immune cells and ductal cells in the metastatic microenvironment of PDAC

**Fig. 7:**
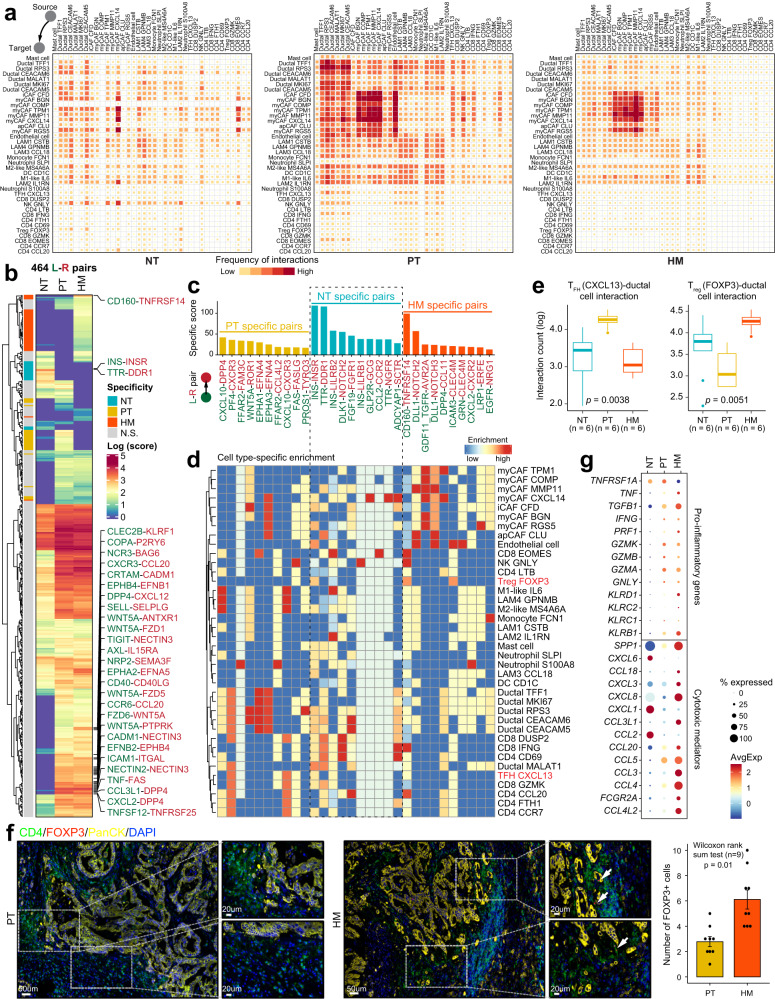
Dynamics of cell–cell interaction networks in the tumor microenvironment of primary and metastatic PDAC tissues. **a** Heatmap illustrating the cell–cell interaction patterns in NT, PT, and HM samples. **b** Heatmap showing the interaction scores for 464 ligand–receptor pairs in NT, PT, and HM groups, respectively. Group specificity is displayed on the left of the heatmap. **c** Bar plot showing group-specific ligand–receptor pairs in three sample groups. **d** Heatmap showing the enrichment of sample-specific ligand–receptor pairs in cell subtypes. **e** Boxplot indicating cell–cell interaction counts between T_FH_ (*CXCL13*) and ductal cells, as well as between T_reg_ (*FOXP3*) and ductal cells. The number of ligand–receptor pairs (*n* = 6) in each category is indicated below the boxplot. The boxes showing the median (horizontal line), second to third quartiles (box), and Tukey-style whiskers (beyond the box). The *p* value is calculated with one-sided Wilcoxon rank-sum test. **f** Immunofluorescent staining showing co-localization of *CD4* (green), *FOXP3* (red), PanCK (yellow), and DAPI (blue) in PT and HM samples. Scale bars of each group, 50 μm (left) and 20 μm (right). The bar plots show the quantification results, *n* = 3 (9 view fields in total). The error bar indicates standard error of the mean (s.e.m.). The *p* value is calculated with one-sided Wilcoxon rank-sum test. **g** Dot plot illustrating the expression and frequency of representative pro-inflammatory and cytotoxic-mediator genes in NT, PT, and HM samples.

Finally, to decipher the crosstalk between tumor cells and other components in the tumor microenvironment during PDAC metastasis, we utilized a public repository of ligand–receptor (L–R) interactions, CellPhoneDB^34^, to visualize L-R-mediated intercellular interactions. In general, the interaction among different cell types was much lower in HM tissues than in PT tissues (Fig. 7a). In particular, the interaction between immune cells, especially NK/T cells and other cell types, was significantly reduced in HM tissues compared with PT and NT tissues (Supplementary Fig. 13a, b). Strikingly, we found that NK/T cells barely interacted with tumor ductal cells in HM tissues; the interactions between myeloid cells and ductal cells were also significantly decreased in HM tissues compared with PT tissues (Fig. 7a), suggesting that metastatic tumor cells had less contact with its environment, which might be an important mechanism responsible for the colonization of PDAC cells in the metastasis site.

We identified a total of 464 L-R pairs whose interacting frequencies were significantly different across the three tissue types (Fig. 7b). Notably, the interactions between CAFs and other cell types were more frequently present in HM tissues than in PT and NT tissues (Fig. 7c, d). For example, we identified increased communication between DLL1 and NOTCH2 signals in HM tissues, especially in CAFs (Fig. 7c, d and Supplementary Fig. 14a). On the contrary, we observed less interaction between ductal cells and other cell types, including iCAFs and T cells, in HM tissues compared with PT tissues (Fig. 7c, d). For instance, the crosstalk between WNT5a and ROR1 was only identified in PT-specific ductal cells and iCAFs (Fig. 7c, d and Supplementary Fig. 14b). In addition, a co-expression pattern was identified for PF4/CXCR3 between ductal cells and T cells in PT tissues, suggesting a potential mechanism by which CXCR3^+^ T cells could be recruited to the tumor epithelium. However, the crosstalk between ductal cells and most subtypes of T cells was almost absent in the metastatic microenvironment. The only exception is the FOXP3^+^ T_reg_ cells, which displayed a significantly higher level of interaction with ductal cells in HM tissues than that in PT tissues, highlighting the crucial role of T_reg_ cells in promoting the metastasis of PDAC (Fig. 7e). This was confirmed by immunofluorescence staining in patient samples, showing the enhanced accumulation of CD4^+^/FOXP3^+^ T_reg_ cells surrounding the epithelium in HM tissues (Fig. 7f). Although the FOXP3^+^ T_reg_ cells could also be detected in PT tissues, most of them were not adjacent to the epithelium, suggesting the lack of interaction between T_reg_ cells and ductal cells in PT tissues. In contrast, the interaction between CXCL13^+^ T_FH_ cells and ductal cells was more apparent in PT tissues, which is consistent with other T cell populations (Fig. 7e). Furthermore, HM tissues showed increased expression of a series of chemokine-encoding genes such as CCL18, CCL20, and CXCL8, which were known to inhibit CD8^+^ T cell infiltration and promote the recruitment of T_reg_ cells to bring about an immunosuppressive microenvironment^30,35–37^ (Fig. 7g). Together, these data provide mechanistic insights, from the perspective of intercellular interactions, into the formation of the TME in PDAC patients with liver metastasis.

## Discussion

Despite the progression in surveillance and treatment strategies, the survival outcomes of PDAC patients remain poor due to the high incidence of distal metastasis at initial diagnosis, and after surgical resection^38^. The high degree of intra-tumoral heterogeneity and the complex TME rich in desmoplastic stroma are widely considered the main obstacles to effective treatment of PDAC patients^39^. A recent study showed that primary PDAC tumors and hepatic metastases share high similarities in somatic mutations, copy number variations and transcriptomic profile^12^. However, cellular differences in the TME between primary and metastatic PDAC remain an open question. Up until now, very limited studies have focused on exploring the cellular ecosystems in PDAC metastases using single-cell sequencing approaches^17,18^. Since metastatic tissues could not be acquired through surgical resections in most PDAC patients, these studies only collected limited biopsies from metastases via EUS-FNA, thus hindering the integrated understanding and assessment of the entire tumor microenvironment in metastatic PDAC. In this study, we aim to provide a comprehensive single-cell transcriptomic landscape for characterizing the tumor microenvironment in primary PDAC and matched liver metastases simultaneously using high-quality surgically resected tissues. Our study not only presents a high-resolution description of the cellular diversity in the tumor, stromal, and immune components, but also underlines the intercellular crosstalk in primary and metastatic PDAC.

In the present study, we dissected the cellular composition of both primary PDAC and liver metastases tissues. Neoplastic ductal cells are of high inter-patient heterogeneity in both human and murine PDAC tissues^4,5^ while little is known about the intra-tumoral heterogeneity of metastatic PDAC. Intriguingly, we made an interesting finding that the accumulation of CEACAM5^+^/CEACAM6^+^ ductal cells was associated with poor overall survival in PDAC patients, highlighting the tumor-promoting function of these two ductal cell clusters. CNV-based analysis demonstrated that the proportion of CNV^medium^ and CNV^high^ ductal cells was much higher in HM tissues than PT tissues, confirming the malignant state of metastatic ductal cells. Besides, CNV levels might contribute to the inter-patient heterogeneity of metastatic ductal cells. For example, the percentage of CNV^high^ ductal cells in HM from patient 3 was more than 70%, while CNV^high^ ductal cells constituted less than 10% in HM from patient 1 (Fig. 2c). Recently, Lee et al.^18^ reported that epithelial-mesenchymal transition and hypoxia pathways might lead to the aggressive phenotype of liver metastases using pseudotime analysis in a few biopsy tissues. In our study, we identified five ductal cell states via pseudotime trajectory analysis and further delineated the evolutionary dynamics of pancreatic ductal cell lineages from primary to metastatic PDAC, that is, from a high proliferative but low CNV state in PT tissues to a malignant CNV^high^ state in HM tissues. Importantly, we discovered a panel of malignant genes responsible for PDAC progression and metastasis, such as KLK7, LITAF, and SPRR1B. Interestingly, KLK7, which encodes the kallikrein-related serine peptidase, was recently reported to control the remodeling of tumor microenvironment^40^, validating the functional relevance of our bioinformatics analysis. The functions of other identified genes in the progression of pancreatic cancer are worth investigating in future studies.

CAFs have been shown to play critical roles in promoting tumor growth and metastasis in multiple cancer types^41,42^. Previous studies have identified various CAF subpopulations in both human and murine pancreatic tumors, including myCAF, iCAF, and apCAF^4^. A recent study proved the existence of all three CAFs in biopsy tissues from metastatic PDAC patients; however, they did not investigate how different CAFs contribute to PDAC metastasis at single-cell level^18^. Another recent study demonstrated that the major CAF subtype within hepatic metastases appeared to have a ‘pericyte-like’ expression profile similar to myCAF signatures, suggesting the pathophysiologic features of CAFs were different between the primary and metastatic microenvironment^43^. Yet the molecular events underpinning the function of CAFs in the metastatic microenvironment of PDAC remain poorly defined. In the current study, we validated the accumulation of myCAF, iCAF, and apCAF in both PT and HM tissues, the majority of which were myCAFs. Surprisingly, we identified a subpopulation of myCAF (RGS5^+^) predominantly expressed in HM tissues which was validated by immunofluorescence staining. RGS5^+^ myCAF has been found in bladder cancer^44^ but has not been linked to PDAC. Importantly, pseudotime trajectory analysis revealed RGS5^+^ myCAF as the most mature and malignant CAF population in the metastatic microenvironment, possibly arising from the CFD^+^ iCAF cells. Therefore, the RGS5^+^ myCAF subpopulation could be a potential therapeutic target for advanced PDAC patients. The precise role of RGS5^+^ myCAF in PDAC progression needs to be further explored.

The predominance of myeloid cells in HM tissues prompted us to elaborate the function of myeloid cells in the metastatic microenvironment. We identified six clusters of macrophages, including four subtypes of lipid-associated macrophages (LAMs). Notably, the LAM3 subpopulation was predominantly presented in HM tissues with high expression of CCL18, which has been reported to exert immune-suppressive functions through inhibiting the production of inflammatory factors^45^. Wu et al.^31^ reported that CCL18^+^ macrophages are enriched in metastatic sites of colorectal cancer patients and exhibit an immune-suppressive phenotype and are sensitive to neoadjuvant chemotherapy, emphasizing the crucial role of CCL18^+^ LAMs in the metastatic microenvironment. Using the scMetabolism pipeline, we found that CCL18^+^ LAM3 cells enriched in HM tissues exhibited the highest metabolic activity. The function of CCL18^+^ LAM3 cells in the metastatic microenvironment of PDAC needs to be investigated in depth.

Neutrophils are key components of the metastatic microenvironment and display heterogeneity in multiple cancers^46,47^. Recently, Wang et al.^48^ showed that a subset of P2RX-negative neutrophils accumulates in PDAC liver metastases, and trigger the evasion of antitumor immunity in the metastatic microenvironment; however, the molecular mechanisms underlying neutrophil infiltration in PDAC hasn’t been elucidated at single-cell level. We identified two clusters of tumor-associated neutrophils, both of which were substantially expressed in HM tissues, especially the S100A8^+^ neutrophils. Of note, CXCL8, a chemokine gene found to be highly expressed in both clusters, was reported to promote the immunosuppressive microenvironment^49^. These findings emphasize the role of certain subtypes of neutrophils in promoting PDAC metastasis.

Resistance to most immunotherapies is a hallmark of PDAC, and previous single-cell-based studies have demonstrated profound immune suppression and T cell dysfunction in PDAC tumor microenvironment^16,50^. In this study, we observed the expression of exhausted and inhibitory checkpoint receptors such as LAG3 in most CD8^+^ T cells, which is consistent with a recent study showing that CD8^+^ T cells in PDAC tissues have pronounced exhaustion signatures^16^. Notably, we also identified a subset of GNLY^+^ NK cells in tumor tissues, and found that the percentage of NK cells was profoundly elevated in HM compared with PT tissues, which is contradictory to previous findings that the number and activity of NK cells decrease as the disease progresses in PDAC patients^51^. One possible explanation is that the GNLY^+^ NK cells exhibited increased expression of the immunosuppressive checkpoint molecule HAVCR2, a potential immunotherapeutic target for metastatic PDAC. An enrichment of T_reg_ cells in tumor-infiltrating lymphocytes is observed in many cancers, hepatocellular carcinoma for instance, leading to an immunosuppressive microenvironment^52^. Yet the role of T_reg_ cells in PDAC progression and metastasis is not well understood. In our analysis, the T_reg_ cells expressed high levels of immune checkpoint genes including CTLA4, TIGIT, ICOS, TNFRSF4, and TNFRSF9, representing both exhausted and co-stimulatory signatures. Interestingly, we found that FOXP3^+^ T_reg_ cells exhibited a remarkably enhanced level of interaction with ductal cells in HM compared with PT tissues through interactome prediction analysis and validation with immunofluorescence staining, whereas the overall interaction between total NK/T cells and ductal cells was almost absent in the metastases, possibly due to the excessive interplay between T_reg_ cells and ductal cells. Our dataset a stage for developing immunotherapeutic strategies against T_reg_ cells for advanced PDAC patients.

Our study has some limitations. Firstly, due to the difficulty of acquiring matched primary and metastasis tissues from PDAC patients via surgical resection, the sample size of this study is limited to four patients. Because the tumor microenvironment of the metastatic multifocal tumors is highly heterogeneous even within one patient, our data may not reflect the entire ecosystem of metastatic PDAC. Besides, the ligand–receptor interaction analysis among different cellular components was derived mostly from transcriptomic predictions. Further confirmation of these predicted interactions by high-dimensional multiplex in situ profiling is required.

In summary, our study provides an in-depth characterization of the transcriptomic and functional phenotypes of the diverse cellular components in the tumor microenvironment of a unique set of matched primary and metastatic PDAC tumor samples. The intra-tumoral crosstalk between tumor and immune cells is also highlighted, facilitating a deeper understanding of the mechanisms underlying the immunosuppressive microenvironment in metastatic PDAC. Although further functional validation of these analyses is warranted, our dataset can serve as a valuable resource for the design of targeted therapies and immunotherapeutic approaches for advanced PDAC.

## Methods

All studies comply with all relevant ethical regulations. All research protocols involving human samples were subjected to review by the Ethics Committee of the First Affiliated Hospital of Soochow University and Nanjing Drum Tower Hospital, and received approval for the study protocols as described in detail below.

### Sample collection and preparation

Four patients who were pathologically diagnosed with PDAC with liver metastases were enrolled into this study. None of the patients received any antitumor therapy prior to surgery. Three of the patients (P1–P3) underwent primary PDAC resection, as well as synchronous oligometastatic resection at the First Affiliated Hospital of Soochow University, China. The decision for the operations was made based on extremely careful preoperative assessment of the high-resolution abdominal magnetic resonance imaging (MRI) and enhanced computed tomography (CT) data. The biopsy of the liver metastasis from the fourth patient (P4) was obtained via EUS-FNA operation in Nanjing Drum Tower Hospital Affiliated to Nanjing University Medical School. The patients included males and females, aged 49-73, as the information on sex and gender was not relevant in our study. The clinical characteristics of the patients were shown in Supplementary Table 1. All patients provided written informed consent for sample collection and data analyses prior to operation.

### Tissue dissociation

Tissues were cut into 2-4 mm^3^ segments and underwent cell lysis using gentleMACS (130-093-235; Miltenyi Biotec Germany) and MACSmix (130-090-753; Miltenyi Biotec Germany) in the mixed enzyme solution (4.7 mL Medium, 200 µL Enzyme H, 100 µL Enzyme R, and 25 µL Enzyme A). The lysate was resuspended and filtered through a 70-µm cell strainer (130-098-462; Miltenyi Biotec Germany). Cells were collected by centrifuging (300 × *g* for 7 min at 4 °C) and resuspended at 700–1200 cells/µl. The entire mixed cell populations were analyzed further without sorting or enriching for particular cell subtypes.

### Whole-exome sequencing

Whole-exome capture was performed using an Agilent SureSelect Human All Exon V6 kit. Exome sequencing data were aligned to the GRCh38 human reference genome with BWA (version 0.7.15)^53^. After sorting aligned reads using samtools (version 1.9), we used GATK (version 4.3.0.0) to mark and remove duplicate reads and perform base mass recalibration. The MuTect2 algorithm^54^ was used to identify somatic mutations. MuTect2 identifies candidate somatic mutations by Bayesian statistical analysis of bases and their qualities in the tumor and normal BAMs at a given genomic locus. Variants with mutation allele frequency (MAF) > 0.7 were filtered. Allelic copy numbers in exome sequencing data were estimated using Sequenza with the default options. The variant allele frequency (VAF) of KRAS mutations is defined as the number of variant reads divided by the number of total reads, reported as a percentage. And the VAF for KRAS mutations of the three detected samples were 0.25 for P2-HM, 0.357 for P3-PT and 0.364 for P3-HM separately.

### 10x single-cell sequencing

Cell suspension was loaded to the Chromium Single-Cell v2 3′ and 5′ Chemistry Library, catching 5000–10,000 cells position. Library construction was sequenced on the NextSeq 500 platform (Illumina), receiving 26, 8 and 98 cycles run for Read 1, i7 index and Read 2. All steps were performed according to the manufacturer’s standard protocol.

### scRNA-seq data processing, cluster annotation and data integration

The 10x Chromium single-cell RNA sequencing (scRNA-seq) data were processed using CellRanger (v3.1.0; 10x Genomics) for alignment, barcode assignment and unique molecular identifier (UMI) counting (using the genome reference set GRCh38-3.0.0). Filtered count matrices were converted to sparse matrices using the Seurat package (v3.2.3)^55^, and cells expressing less than 200 genes as well as with more than 20% mitochondrial reads, were excluded from the downstream analysis. The ‘doubletFinder_v3’ method from the DoubletFinder package (v2.0.3)^56^ was applied for additional cell filtering. Filtered data were then log normalized and scaled, with cell–cell variation due to UMI counts and percent mitochondrial reads regressed out.

To avoid batch effects among samples and experiments, integration of single-cell data was performed using Seurat’s canonical correlation analysis (CCA) integration method. A total of 2000 features for anchoring (the ‘FindIntegrationAnchors’ function) and 30 dimensions for alignment (‘IntegrateData’) were used. Cell clustering was performed by ‘FindClusters’ function at a resolution of 0.8 and the top 20 genes were used to define cell identity. Dimensionality reduction was performed with ‘RunUMAP’ function and visualized by Uniform Manifold Approximation and Projection (UMAP). For subgroup cell clustering, cells of different types were extracted separately and clustered by their respective first 30 principal components (PCs) using different resolutions based on visual inspection.

### Identification of signature genes

We applied the ‘FindAllMarkers’ function in Seurat to identify specific genes for each cell subset. For the selection of marker genes specific to each cell cluster/subset, we calculated the log2 fold change (log2FC) between two groups (a cell cluster/subset vs. other cells) using the ‘FindMarkers’ function with the Wilcoxon rank-sum test (default parameters).

### CNV estimation in ductal cells

The InferCNV package (version 1.6.0)^23^ was used to infer CNVs in EPCAM^+^ ductal cells and to recognize cancer cells with default parameters. The CNV signal for individual cells was estimated with a 100-gene sliding window. Genes with a mean count of less than 0.1 across all cells were filtered out before analysis and the signal were denoised using a dynamic threshold of 1.3 s.d. from the mean. Ductal cells were divided into four groups based on CNV accumulation scores: normal, low, medium, high in the analysis.

### Gene signatures

Inflammation-associated genes, including IFNG, IFNGR1, IFNGR2, IL10, IL12A, IL12B, IL12RB1, IL12RB2, IL13, IL17A, IL17F, IL18, IL18R1, IL18RAP, IL1A, IL1B, IL2, IL21, IL21R, IL22, IL23A, IL23R, IL2RG, IL4, IL4R, IL5, IL6, JUN, NFKB1, RELA, RORA, RORC, S100A8, S100A9, STAT1, STAT3, STAT4, STAT6, TGFB1, TGFB2, TGFB3, and TNF, were obtained from previous publication by Smillie et al.^57^.

The cytotoxic gene list consists of 12 genes that translate to effector cytotoxic proteins (GZMA, GZMB, GZMH, GZMK, GZMM, GNLY, PRF1, and FASLG) and well-described cytotoxic T cell activation markers (IFNG, TNF, IL2R, and IL2). The list of genes used for dysfunctional T cells were obtained from Li et al.^58^ and the TAM gene list from Cassetta et al.^59^. Clinically targetable receptor or ligand immune modulator markers expressed on the surface of cells were taken from Wu et al. ^28^.

### Pathway analysis

Differentially expressed genes (DEGs) were detected by the ‘FindAllMarkers’ function in Seurat, using |FC| > 2 and adjusted *p* value < 0.05 as the cut-off values. GO enrichment analysis on DEGs in this study were performed by the clusterProfiler package^60^. GSVA was conducted using the GSVA package^61^. Differences between different cell groups were calculated by the ‘FindMarkers’ function in the Seurat package.

### Pseudotime analysis by Monocle

Ductal cell and CAF cell developmental trajectories were inferred using Monocle2 (version 2.99.3)^25^ with default parameters as recommended by the developers. Firstly, integrated gene expression matrices from specific cell type were exported from Seurat into Monocle to construct a CellDataSet. Secondly, the ‘setOrderingFilter’ function was applied to sort cells with the variable genes identified by the function of ‘differentialGeneTest’ (cutoff of *q* < 0.001). Finally, after dimensionality reduction using the ‘reduceDimension’ function (using the ‘DDRTree’ reduction method), a series of representative key role genes were revealed along the differentiation progress by the ‘plot_pseudotime_heatmap’ function. Dimensionality reduction was performed with no normalization and the ‘DDRTree’ reduction method in the ‘reduceDimension’ step. The visualization function “plot_cell_trajectory” were used to plot each group along the same pseudotime trajectory.

### RNA velocity

We used scVelo^62^ (version 0.2.3) to calculate the single-cell trajectory/directionality using spliced and unspliced reads from the pre-aligned bam files. From loom files produced by the command-line tool, we subset the exact same cells that were previously selected for Monocle trajectory analysis. RNA velocity, latent time, root, and terminal states were calculated using the dynamical velocity model.

### Cell–cell interaction analysis

Cell–cell interactions among the cell types were estimated by CellPhoneDB (v2.1.1)^34^ with default parameters (20% of cells expressing the ligand/receptor) and using the version 2.0.0 of the database. CellPhoneDB infers the potential interaction strength between two cell subsets based on the gene expression level of a receptor-receptor pair. The significance of interaction is assessed through a permutation test (1000 times). The normalized gene expression was used as input. Interactions with *p* value < 0.05 were considered significant. We only considered ligand–receptor interactions based on the annotation from the database, for which only and at least one partner of the interacting pair was a receptor, thus discarding receptor-receptor interactions and other interactions not involving a receptor.

### Correlation to public datasets

Using the Cancer Genome Atlas (TCGA) PDAC bulk RNA-seq datasets, the relative abundance of subtypes of ductal cells was predicted by CIBERSORTx^21^ algorithm (https://cibersortx.stanford.edu/) with default parameters. The tumor samples were divided into two groups based on the estimated relative cell abundance. Overall survival analysis was performed with Cox proportional hazards regression using ‘coxph’ from the R package *Survival*. Kaplan-Meier plots were used to assess the prognostic value of cell types and to explore their effect in PDAC cancer progression.

### Survival analysis

RNA-seq and clinical data of PDAC samples were obtained from TCGA to evaluate the prognostic effects of gene sets derived from specific cell states. To assess the impact of specific differentially expressed marker genes on PDAC cancer progression, the tumor samples were divided into two groups with high 50% and low 50% of the mean expression of the target genes. Survival curves were performed by the Kaplan–Meier method with the *Survival* package v.2.44, and visualized using the ‘ggsurvplot’ function of the *survminer* package. Significance was assessed by the log-rank test statistics (*p* values) between two groups.

### Immunohistochemistry (IHC) staining

Formalin-fixed, paraffin-embedded PDAC and matched liver metastasis specimens were obtained from Nanjing Drum Tower Hospital (*n* = 11) and the First Affiliated Hospital of Soochow University (*n* = 10) for IHC staining of LITAF protein. All the specimens were pathologically validated as PDAC with liver metastasis. Besides, another cohort containing 46 PDAC tissues (PT) and adjacent normal pancreas tissues (NT) was obtained from Nanjing Drum Tower Hospital for IHC staining of LITAF. Ethical approval was obtained from the ethics committee of Nanjing Drum Tower Hospital and the First Affiliated Hospital of Soochow University.

IHC staining was performed as described previously^63^. Slides of the specimens were sectioned at 4-μm thickness, deparaffinized, blocked, and incubated overnight at 4 °C with primary antibody, followed by horseradish peroxidase-labeled secondary antibody Goat Anti-Rabbit IgG H&L HRP (1:2000, ab205718, Abcam) at room temperature for 2 h. The primary antibody used in this study was LITAF (1:100, 16797-1-AP, ProteinTech).

### Multiplexed immunofluorescence (IF) staining

Formalin-fixed, paraffin-embedded PDAC and matched liver metastasis specimens were obtained from the First Affiliated Hospital of Soochow University. Multiplexed IF staining was performed for the 3 pairs of PDAC and liver metastasis samples (paired PT and HM) undergoing scRNA-seq using the PANO 5-plex IHC kit (Cat# 10002100100, Panovue, Beijing, China) according to the manufacturer’s instructions. Multiplexed IF staining was performed for the analysis of S100A8^+^ neutrophils using 10 pairs of matched samples.

Multiple primary antibodies, including RGS5 (1:300, 11590-1-AP, Proteintech), ACTA2/smooth muscle actin (1:500, 14395-1-AP, Proteintech), Pan Cytokeratin (1:500, 53-9003-82, Invitrogen), FN1 (1:200, 66042-1-Ig, Proteintech), S100A8 (1:200, 15792-1-AP, Proteintech), MPO (1:1000, ab208670, Abcam), CD4 (1:500, 67786-1-Ig, Proteintech), CD8 (1:1000, 66868-1-Ig, Proteintech), CD68 (1:400, 76437, CST), CCL18 (1:200, 22303-1-AP, Proteintech), FOXP3 (1:500, ab20034, Abcam) and GNLY/Granulysin (1:1000, ab241333, Abcam), were used in this study, followed by incubation with horseradish peroxidase-conjugated secondary antibodies and tyramide signal amplification. The slides were microwave heat-treated after each tyramide signal amplification operation. Nuclei were stained with DAPI after all the antigens above had been labeled. The stained slides were imaged at ×10 magnification using a Vectra 3.0 Automated Quantitative Imaging System (Perkin Elmer) and regions of interest (ROIs) were selected for multispectral image acquisition at ×20. Five high-power fields were taken per patient sample to quantitate the average number of certain cell populations.

### Reporting summary

Further information on research design is available in the Nature Portfolio Reporting Summary linked to this article.

### Supplementary information

Supplementary Information

Description of Additional Supplementary Files

Supplementary Data 1

Supplementary Data 2

Supplementary Data 3

Supplementary Data 4

Supplementary Data 5

Supplementary Data 6

Supplementary Data 7

Reporting Summary

### Source data

Source Data

## Data Availability

The processed scRNA-seq data generated in this study are available through the Gene Expression Omnibus under accession number GSE197177. The raw sequence data generated in this study have been deposited in the Genome Sequence Archive (GSA-Human: HRA004556 (scRNA-seq); GSA-Human: HRA004625 (WES)) and are publicly accessible at https://ngdc.cncb.ac.cn/search/?dbId=hra&q=HRA004556 (scRNA-seq) and https://ngdc.cncb.ac.cn/search/?dbId=hra&q=HRA004625 (WES). The publicly available datasets reused in this study, including Peng *et al*.’s dataset^5^ (including 11 NT and 14 PT samples) was retrieved from the Genome Sequence Archive under the accession number GSA: CRA001160 and Yang et al.’ dataset^12^ (including 6 NT, 13 PT and 14 HM samples) was retrieved from GSE151580. The inflammation-associated genes (Fig. 1b) were obtained from previous publication by Smillie et al. ^57^, whose data were deposited in Single Cell Portal: SCP259. The TCGA PAAD dataset was obtained from GDC data portal (https://portal.gdc.cancer.gov/projects/TCGA-PAAD). Source data are provided with the paper. The remaining data are available within the article, Supplementary Information or Source Data file. Source data are provided with this paper.

## Code availability

R codes used to analyze data and generate figures are available at Github (https://github.com/compbioNJU/scPDAC).
