# Supplementary material for: Single cell transcriptomic analyses implicate an immunosuppressive tumor microenvironment in pancreatic cancer liver metastasis
Source: Nat Commun. 2023 Aug 23;14:5123. doi: 10.1038/s41467-023-40727-7 (PMC10447466; doi:10.1038/s41467-023-40727-7)
Supplement: Supplementary file 1 — Supplementary Information [file 41467_2023_40727_MOESM1_ESM.pdf]

Supplementary Information for "Single cell transcriptomic analyses implicate an immunosuppressive tumor microenvironment in pancreatic cancer liver metastasis"

by Zhang et al.

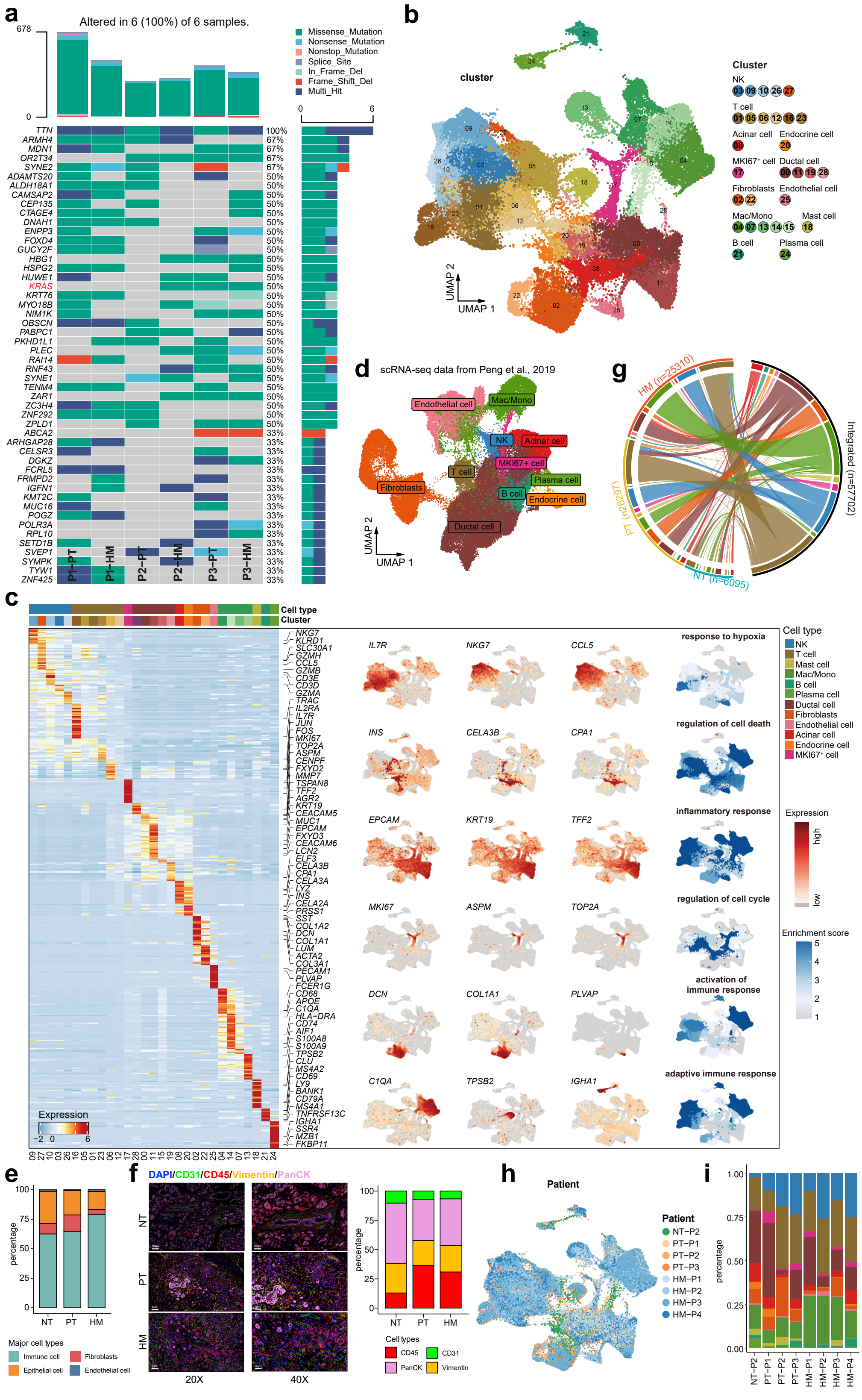

Supplementary Fig. 1. Comprehensive dissection and clustering of 57,702 single cells from primary and metastatic PDAC tissues. Related to Figure 1. **(a)** Top 50 mutated genes called from WES data in three patients with matched PT and HM tissues. **(b)** UMAP plot of 57,702 cells colored by the cell clusters. **(c)** Heatmap showing the scaled expression level of cell subset-specific marker genes (left). Examples of marker gene expression are shown in the middle UMAPs and the enriched GO terms of biological processes for highly expressed genes in each cell type are shown in the right UMAPs. **(d)** UMAP plot displaying the integrated cell map, which consists of 11 cell types from scRNA-seq datasets (n=25) for primary pancreatic tumors (Peng et al., 2019[1]). Cells are colored by cell types. **(e)** Bar plot showing the percentage of cells in each group across all major cell types. **(f)** Immunofluorescent staining showing co-localization of CD31 (green), CD45 (red), Vimentin(yellow), PanCK (purple) and DAPI (blue) in NT, PT and HM samples. Scale bars, 55μm (left) and 25μm (right). This experiments was repeated in 3 patients with similar results. Source data are provided as a Source Data file. **(g)** Chord diagram displaying the relationship of cell types annotated in the integrated cell map in each sample groups and all samples as a whole. The width of the line represents the proportion of cell types. **(h)** UMAP plot of 57,702 cells colored by the cell clusters/subsets (left) or by samples (right). **(i)** Bar plot representing the percentage of cells for each major cell type in different samples.

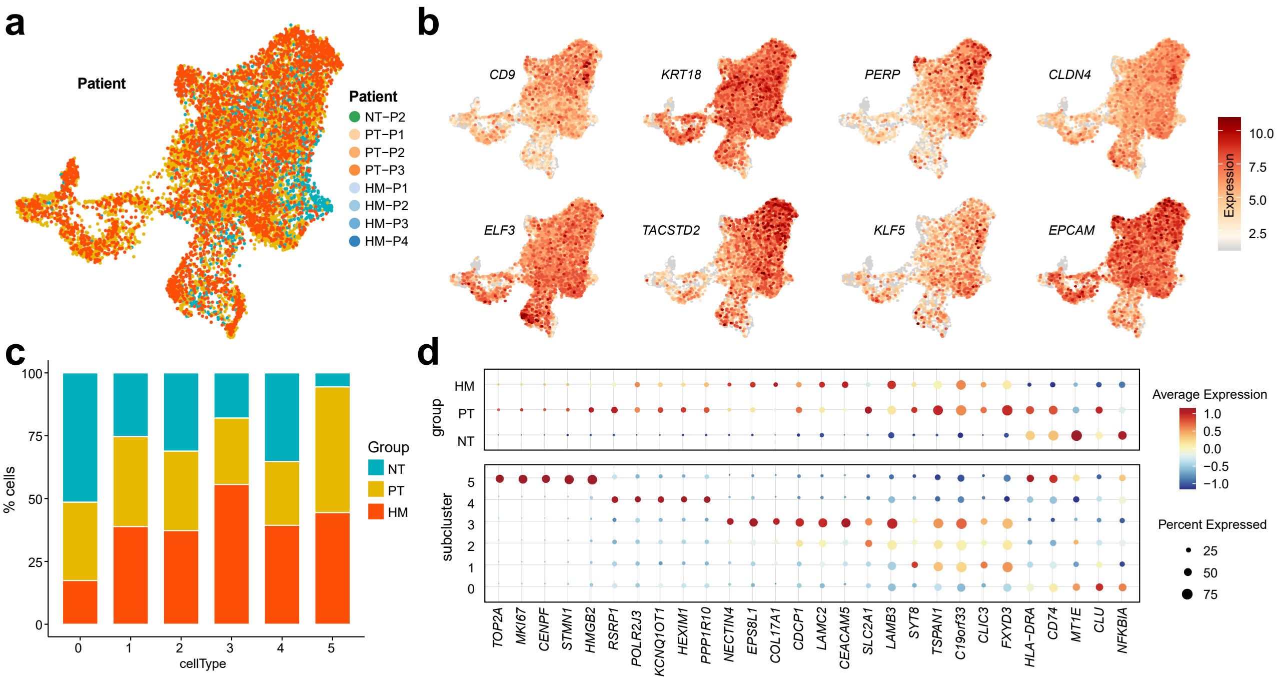

Supplementary Fig. 2. Transcriptional signatures of epithelial cells. Related to Figure 2. **(a)** UMAP showing re-clustering analysis of epithelial cells (ECs), colored by patients. **(b)** UMAP plot showing the expression pattern of eight classical EC marker genes. **(c)** Bar plot showing the percentage of cells in each sample group across all EC subtypes.**(d)** Dot plots showing expression of the top five EC-subtype-specific gene markers in sample groups (top) or cell subtypes (bottom).

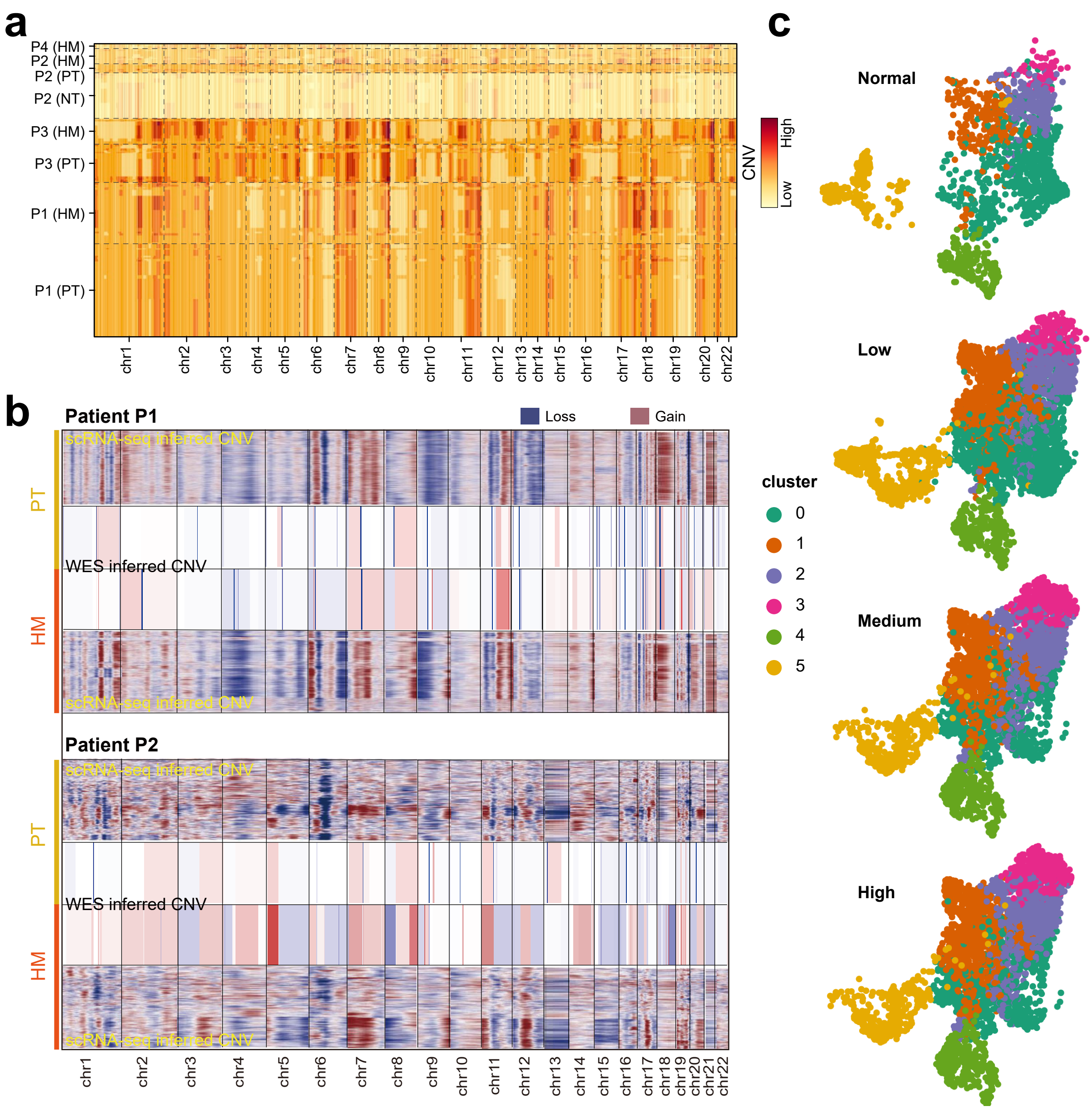

Supplementary Fig. 3. Copy Number Variation analysis of epithelial cells. Related to Figure 2. **(a)** Heatmap showing the predicted CNV level in the eight samples. The normalized CNV levels were shown, the red color represents high CNV level and yellow represents low CNV level. **(b)** CNV inferred by scRNA-seq or WES data in the same patient. **(c)** UMAP showing the distribution of ECs based on CNV normal, low, median and high. Cells are colored according to the subtype.

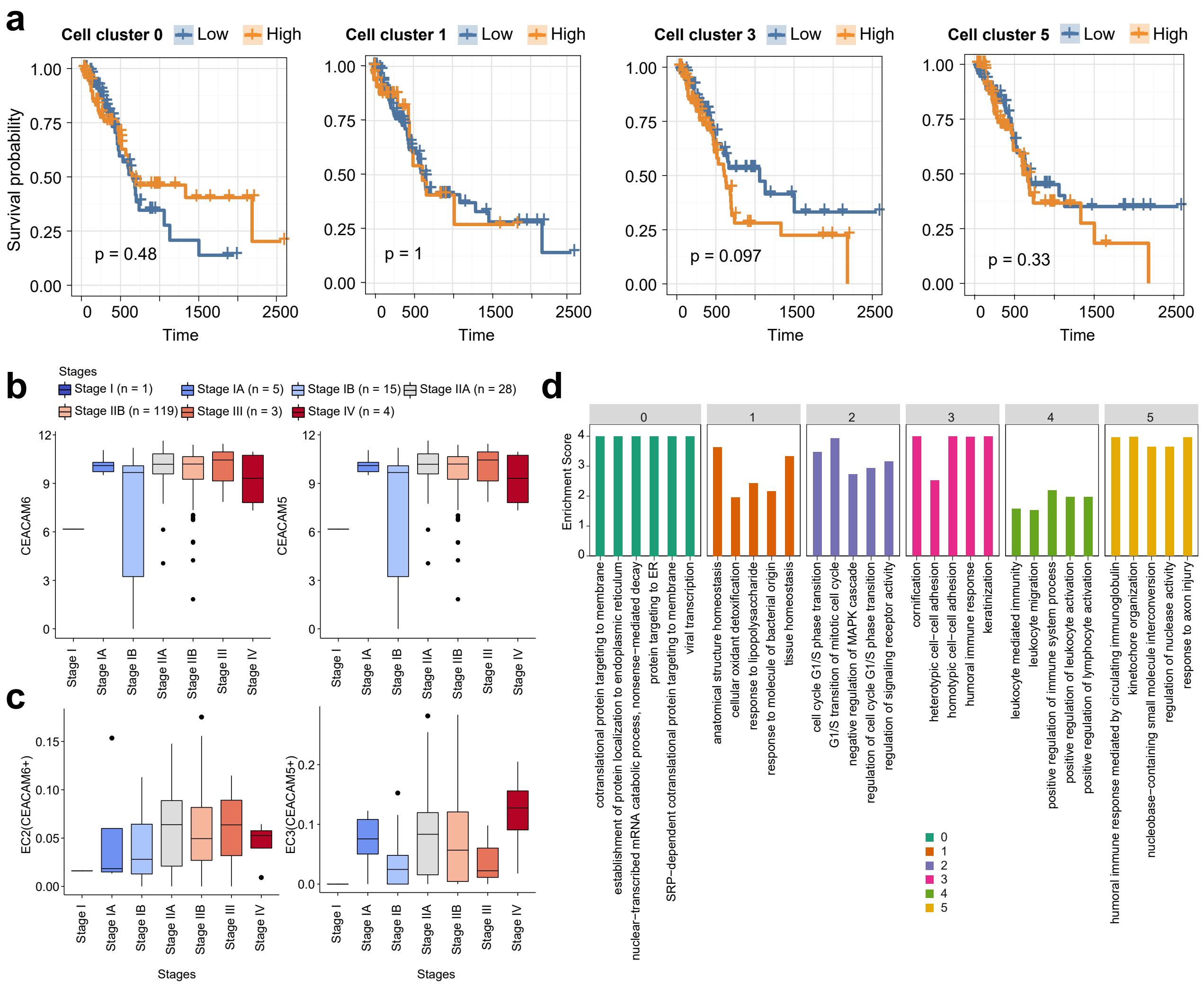

Supplementary Fig. 4. Malignancy of epithelial cells. Related to Figure 2. **(a)** Kaplan-Meier curves for TCGA PDAC patients (n = 178) showing the survival rate grouped by the abundance of EC subtypes. The p-value was calculated using the two-sided log-rank test. **(b)** Expression of *CEACAM5* and *CEACAM6* in TCGA PDAC samples (n = 178) with different clinical stages. The number of samples in each stage is indicated in the legend. The boxes showing the median (horizontal line), second to third quartiles (box), and Tukey-style whiskers (beyond the box). **(c)** Inferred cell type abundance in TCGA PDAC samples (n = 178) using CIBERSORTx. The number of samples in each stage is indicated in the legend. The boxes showing the median (horizontal line), second to third quartiles (box), and Tukey-style whiskers (beyond the box). **(d)**Gene set enrichment analysis in each cell cluster.

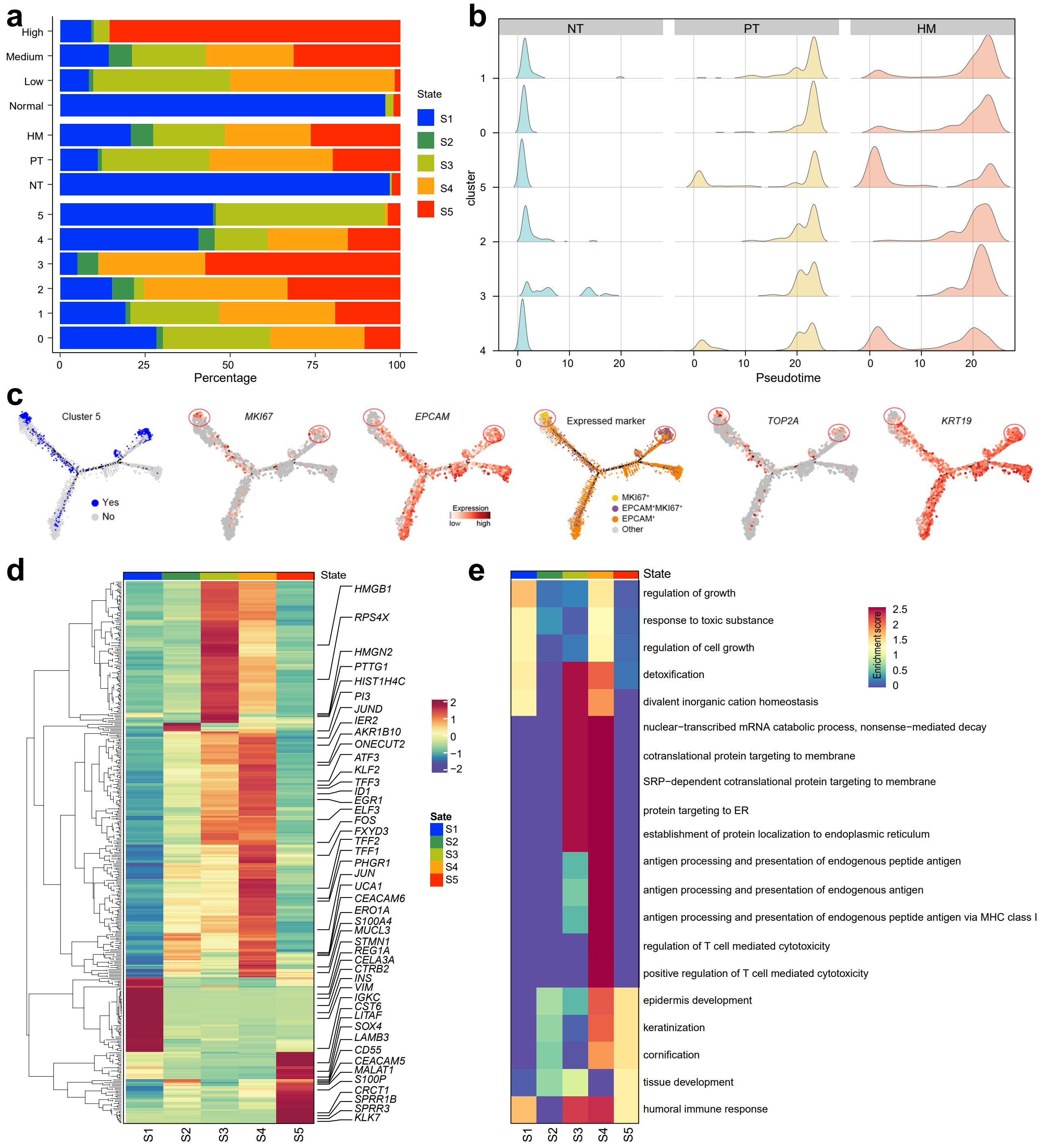

Supplementary Fig. 5. Pseudotime trajectory analysis of epithelial cells. Related to Figure 3. **(a)** Bar plots showing the percentage of cells for each cell state as defined by the pseudotime trajectory based on CNV levels (top), sample groups (middle) or cell subtypes (bottom). **(b)** Diagrams showing the density distribution of cell subtypes in different sample groups along the predicted pseudotime. **(c)** Distribution of marker genes and clusters on branches during pseudotime differentiation trajectory. **(d)** Heatmap visualizing the scaled expression of cell differentially expressed genes in epithelial cells among cell states. Selected marker genes are highlighted. **(e)** Heatmap showing the top five pathways enriched in cell states (S1-S5) by GSEA analyses.

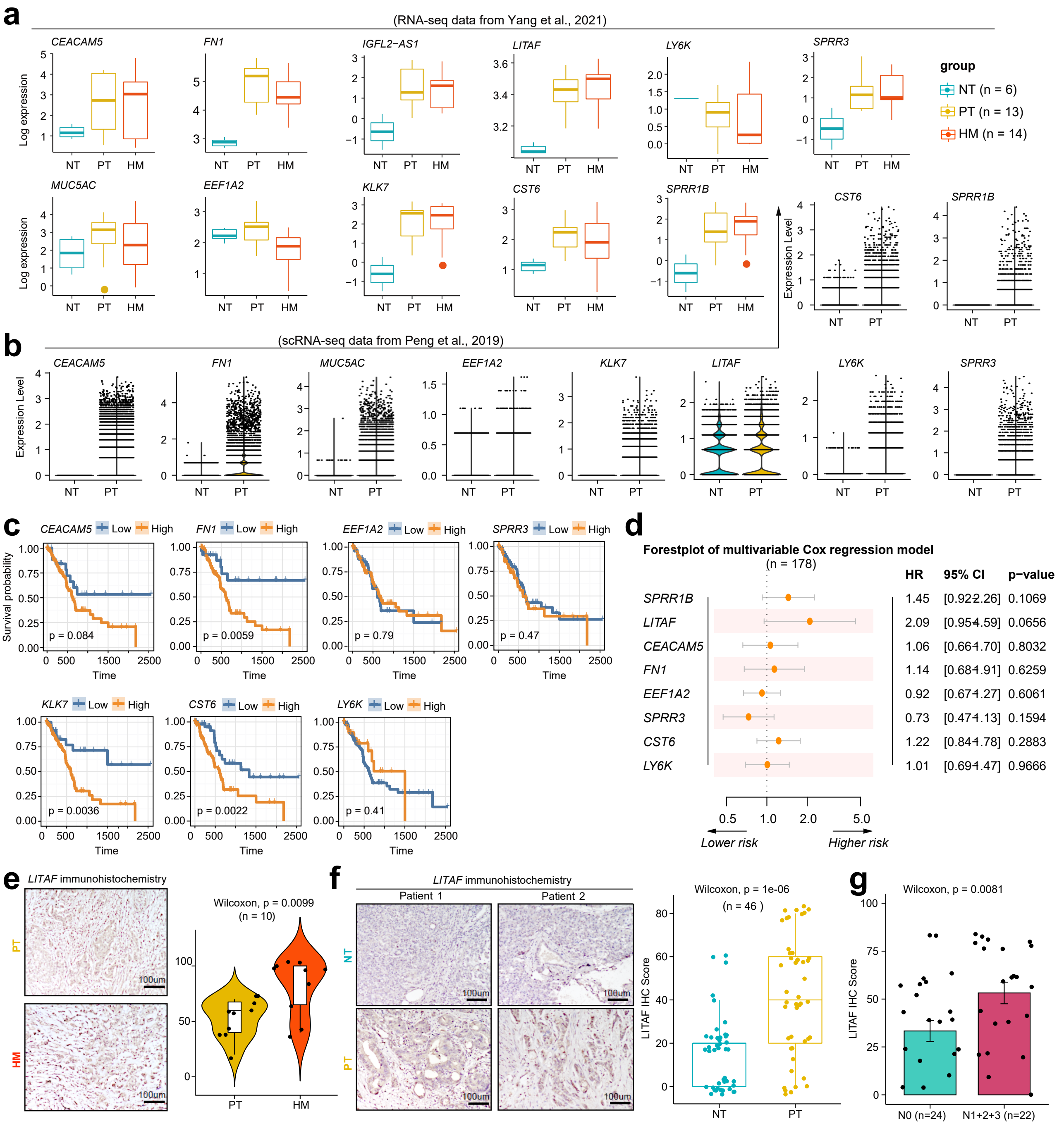

Supplementary Fig. 6. Validations of key findings of epithelial cells in large cohort samples. Related to Figure 3. **(a)** Boxplots showing the expression pattern of differentially expressed genes highlighted in **(Figure 3h)** using the RNA-seq dataset from Yang et al., 2021[2] (NT: n = 6; PT: n = 13; HM: n = 14). The number of samples in each group is indicated in the legend. The boxes showing the median (horizontal line), second to third quartiles (box), and Tukey-style whiskers (beyond the box). **(b)**Violin plots showing the expression pattern of differentially expressed genes highlighted in **(Figure 3h)** using the scRNA-seq dataset from Peng et al., 2019[1]. **(c)** Kaplan-Meier curves of TCGA PDAC patients showing the survival rate grouped by the expression levels of the indicated marker genes. The p-value was calculated using the two-sided log-rank test. **(d)** Forest plot showing the hazard regression of selected genes based on TCGA PDAC data (n = 178). The p-value is calculated with two-sided log-rank test. **(e)** IHC analysis of LITAF in PT and HM samples from the First Affiliated Hospital of Soochow University. Scale bars, 100μm. The bar plot showing the quantification results,n = 10 patients with paired PT and HM samples. The p-value is calculated with one-sided Wilcoxon rank sum test. Source data are provided as a Source Data file. **(f)** IHC analysis of LITAF in NT and PT samples from Nanjing Drum Tower Hospital (n=46 patients). Scale bars, 100μm. The bar plot showing the quantification results, the p-value is calculated with one-sided Wilcoxon rank sum test. The boxes showing the median (horizontal line), second to third quartiles (box), and Tukey-style whiskers (beyond the box). Source data are provided as a Source Data file. **(g)** Bar plot showing the correlation between LITAF expression and lymph node metastasis (n=46 patients). The p-value is calculated with one-sided Wilcoxon rank sum test. The boxes showing the median (horizontal line), second to third quartiles (box), and Tukey-style whiskers (beyond the box). Source data are provided as a Source Data file.

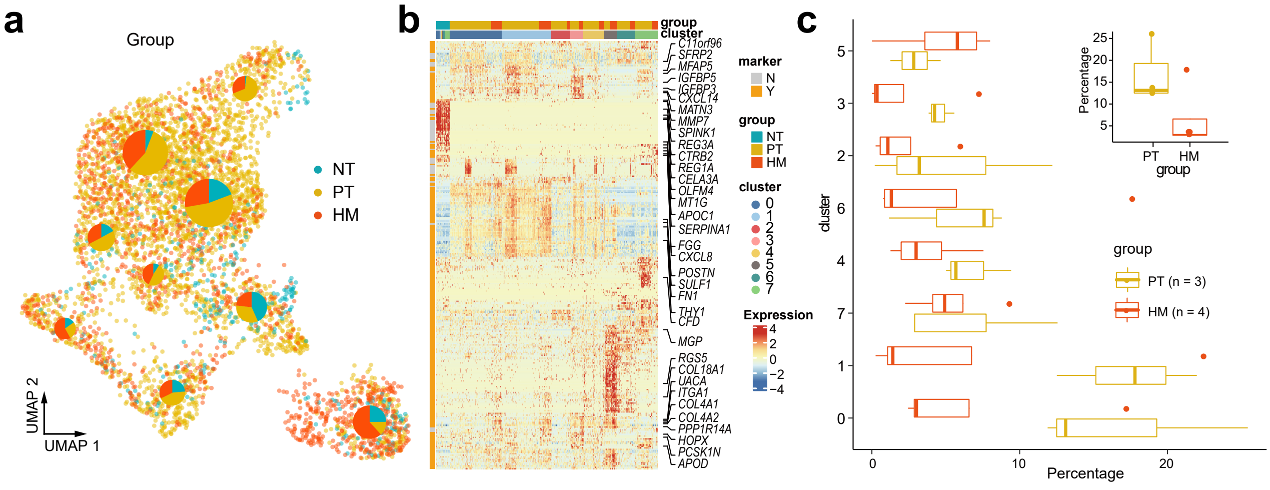

Supplementary Fig. 7. The transcriptional landscape of stromal cells in PDAC microenvironment. Related to Figure 4. **(a)** Distribution of stromal cells in different groups on the UMAP. Pie chart showing the proportion of three sample groups in each cell subtype. **(b)** Heatmap showing the scaled expression levels of cell subtype-specific marker genes. **(c)** Box plots shows the percentage of cells in PT and HM groups for each subtype. The top right box plot showing the percentage of cells in PT and HM groups as a whole. The number of samples in each category is indicated in the left/bottom of boxplot. The boxes showing the median (horizontal line), second to third quartiles (box), and Tukey-style whiskers (beyond the box).

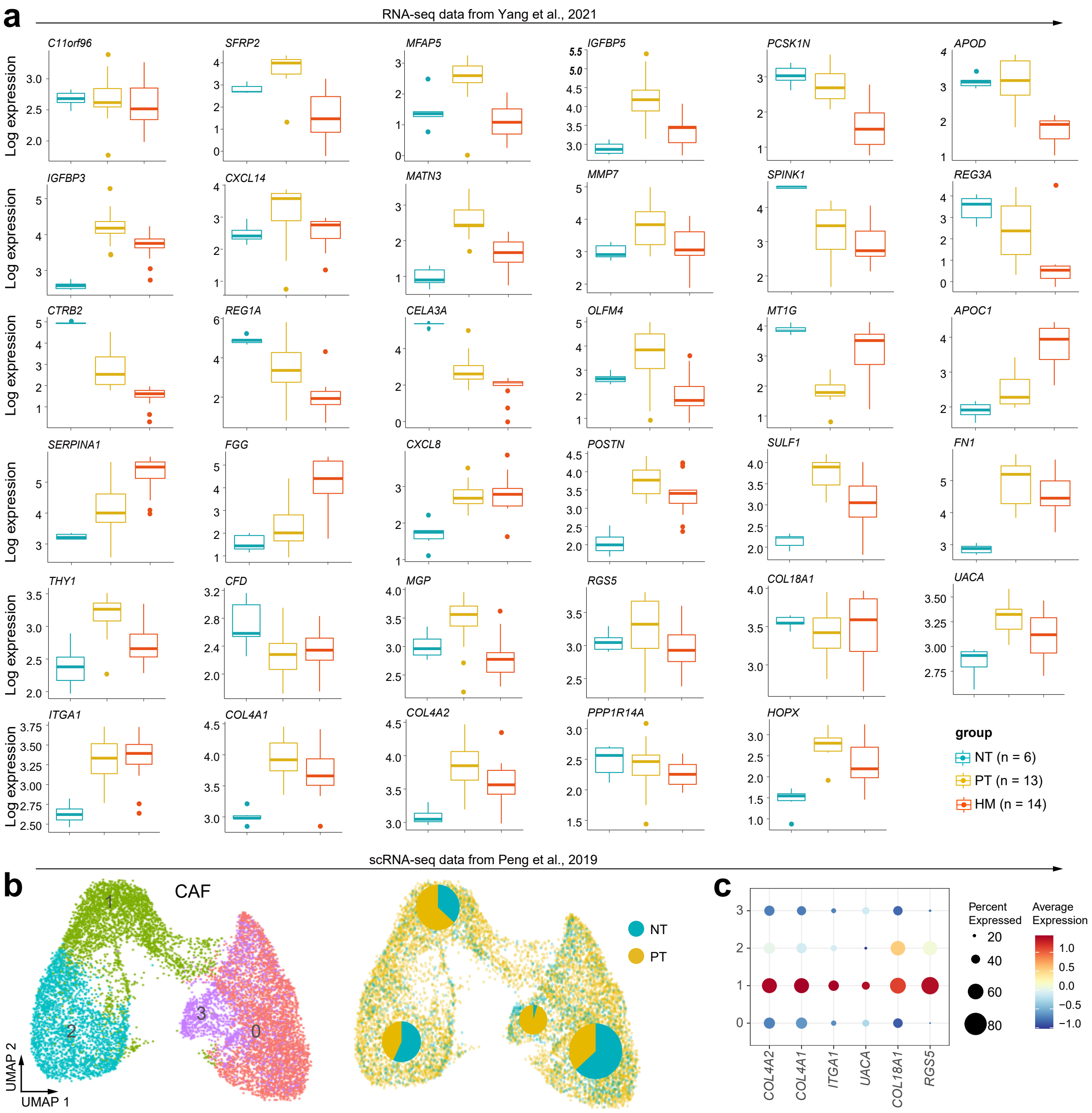

Supplementary Fig. 8. Validations of key findings of stromal cells in large cohort samples. Related to Figure 4. **(a)**Boxplots showing the expression pattern of differentially expressed genes highlighted in Figure 4e using the RNA-seq dataset from Yang et al., 2021[2]. The number of samples in each group is indicated in the legend. The boxes showing the median (horizontal line), second to third quartiles (box), and Tukey-style whiskers (beyond the box). **(b)** UMAP plot of CAFs colored by the cell subsets (left) or by samples (right), scRNA-seq dataset using the Peng et al., 2019[1]. **(c)** Dot plots showing the expression of highly expressed *RGS5+* myCAFs genes in the CAFs subsets, scRNA-seq dataset using the Peng et al., 2019[1]

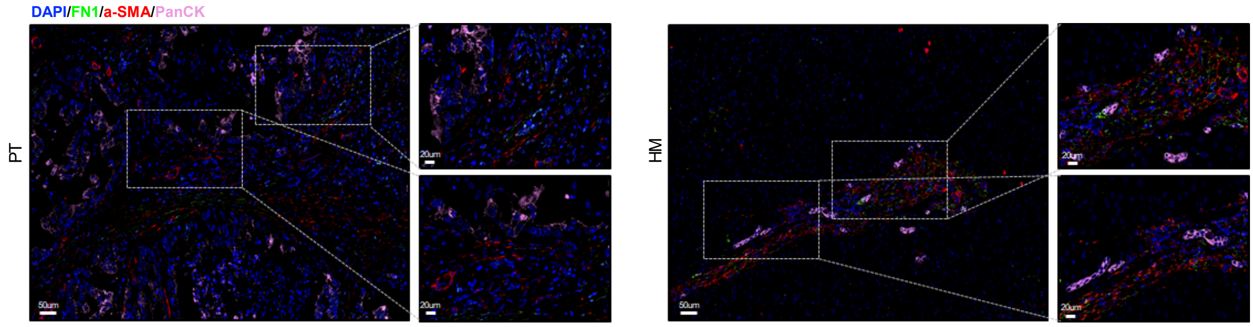

Supplementary Fig. 9. Immunofluorescent staining showing co-localization of FN1 and a-SMA. Related to Figure 4. Immunofluorescent staining showing co-localization of FN1 (green), a-SMA (red), PanCK (purple) and DAPI (blue) in PT and HM samples (n = 3 patients). Scale bars of each group, 50μm (left) and 20μm (right).

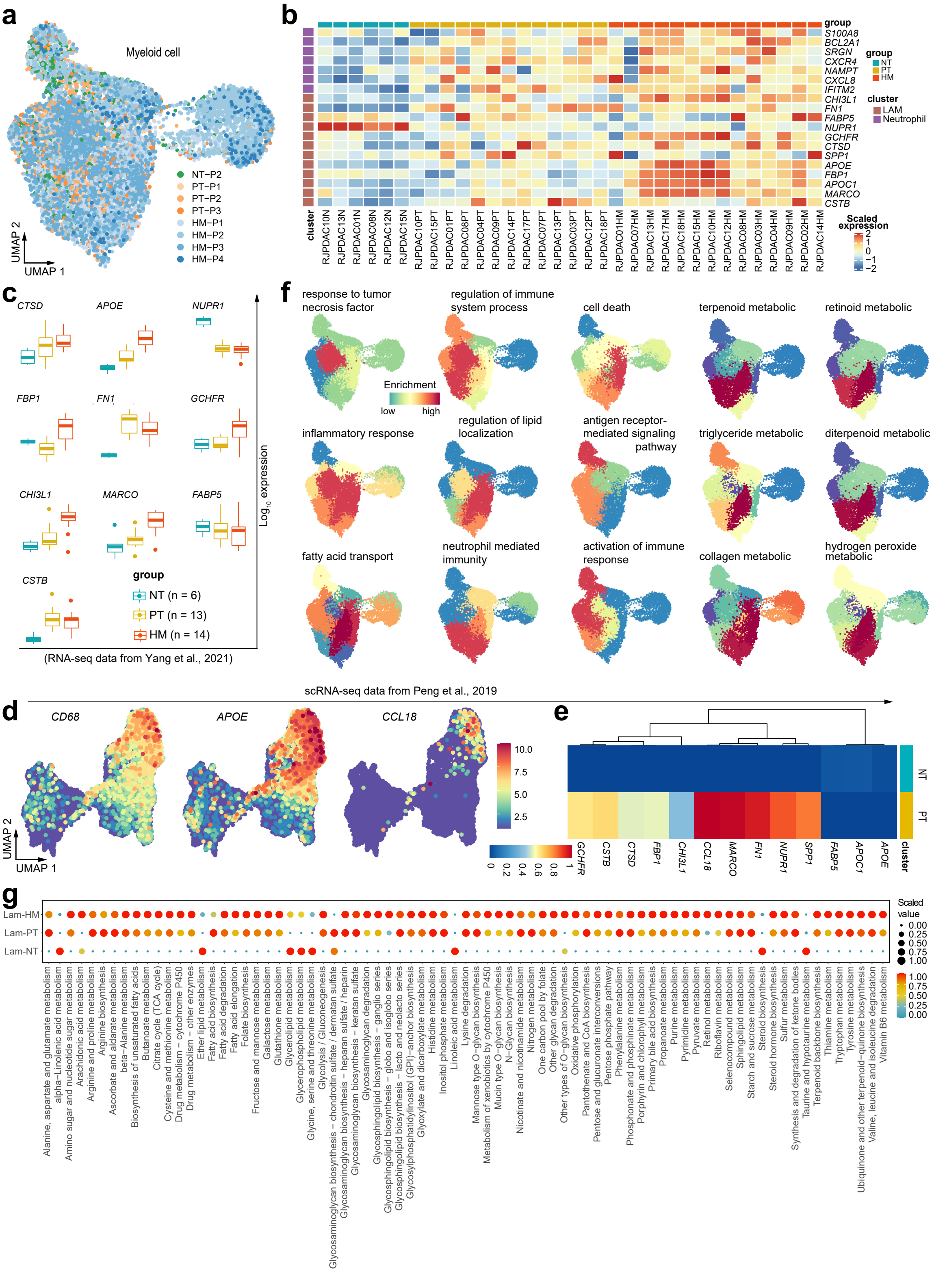

Supplementary Fig. 10. The transcriptional landscape of myeloid cells in PDAC microenvironment. Related to Figure 5. **(a)** Distribution of myeloid cells in different samples on the UMAP. **(b)** Heatmap showing the scaled expression of DEGs in **Figure 5e** using RNA-seq dataset from Yang et al., 2021. **(c)** Boxplots showing the expression patterns of lipid-associated macrophage (LAM)-associated genes in **Figure 5e** using the RNA-seq dataset from Yang et al., 2021[2]. The number of samples in each group is indicated in the legend. The boxes showing the median (horizontal line), second to third quartiles (box), and Tukey-style whiskers (beyond the box). **(d)** Feature plots showing the expression of selected LAM-specific genes. Cells with the highest expression level are colored red, scRNA-seq dataset using the Peng et al., 2019[1]. **(e)** Heatmap showing the scaled expression level of lipid-associated macrophage (LAM)-associated genes in Figure 5e among cells with different groups, scRNA-seq dataset using the Peng et al., 2019[1]. **(f)** UMAPs displaying the enriched GO terms of biological processes for highly expressed genes in each cell subtype. **(g)** The metabolic activity analysis of LAMs revealing that PT and HM groups show overall high metabolic scores. The circle size and color darkness both represent the scaled metabolic score.

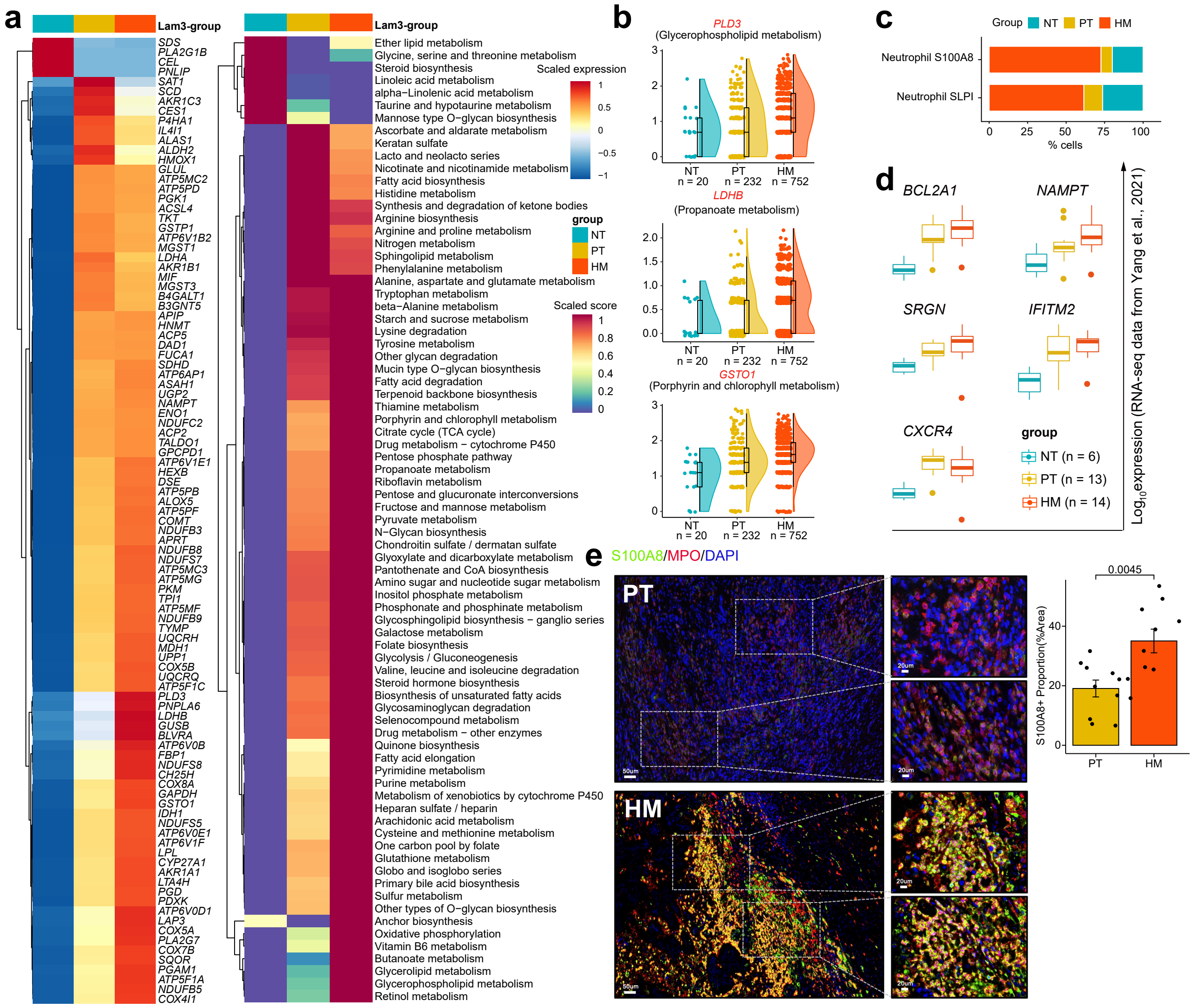

Supplementary Fig. 11. Metabolic characteristics of LAM3 and neutrophils. Related to Figure 5. **(a)** Heatmap showing the scaled expression of metabolic genes (left) and the metabolic pathway score (right) in lipid-associated macrophages 3 (LAM3). **(b)** Violin plot showing the expression of selected metabolic genes. The number of cells in each group is indicated below the violin plot (NT: n = 20; PT: n = 232; HM: n = 752). The boxes showing the median (horizontal line), second to third quartiles (box), and Tukey-style whiskers (beyond the box). **(c)** Bar plot showing the percentage of neutrophils in each sample group. **(d)** Boxplots showing the expression patterns of highly expressed genes in neutrophils using the RNA-seq dataset from Yang et al., 2021[2]. The number of samples in each group is indicated in the legend. The boxes showing the median (horizontal line), second to third quartiles (box), and Tukey-style whiskers (beyond the box). **(e)** Immunofluorescent staining showing co-locations of S100A8 (green), MPO (red) and DAPI (blue) in PT and HM samples. Scale bars, 50μm (left) and 20μm (right). The bar plots show the quantification results, n = 10 patients with paired PT and HM samples. The error bar indicates standard error of the mean (s.e.m.). The p-value is calculated with one-sided Wilcoxon rank sum test. Source data are provided as a Source Data file.

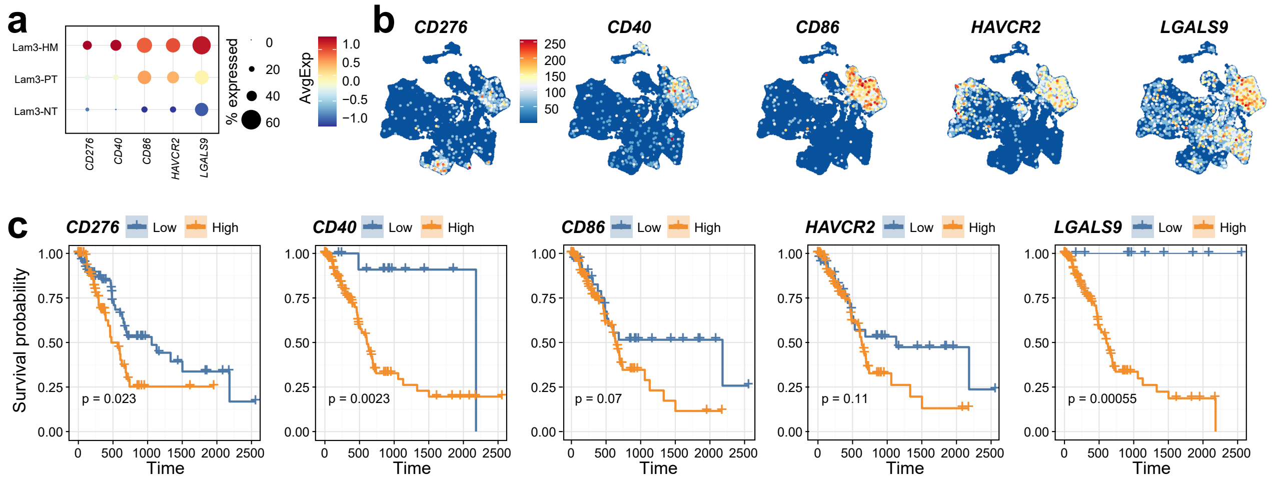

Supplementary Fig. 12. Representative checkpoint genes in LAM3. Related to Figure 5. **(a)** Dot plots showing representative checkpoint genes across sample groups in LAM3. **(b)** UMAPs showing the expression pattern of checkpoint genes from **(a)** in all cells. **(c)** Kaplan-Meier curves for TCGA PDAC patients (n = 178) showing the survival rate in checkpoint genes from **(a)**. The p-value was calculated with the two-sided log-rank test.

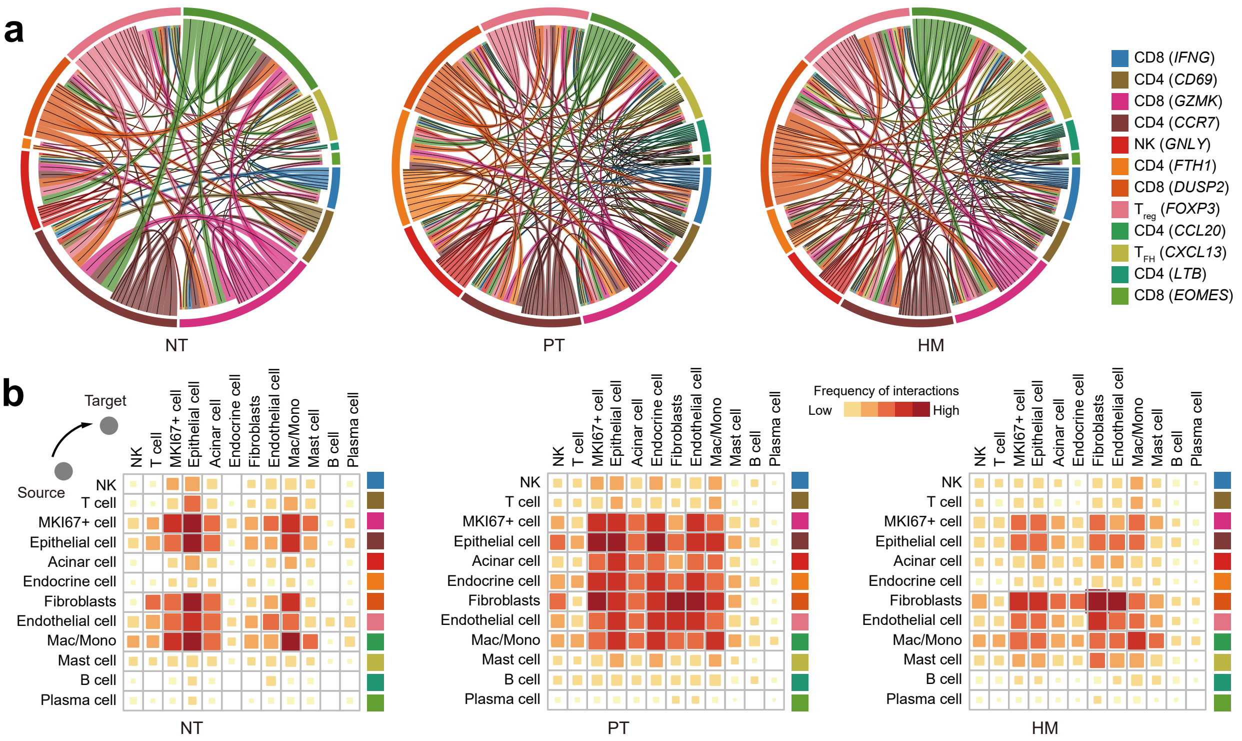

Supplementary Fig. 13. Cell-cell interaction landscape in major cell types. Related to Figure 7. **(a)** Chord diagrams of cell-cell interaction networks among all cell types in different groups. **(b)** Heatmap showing the interaction intensity of cellular interactome from **(a)**.

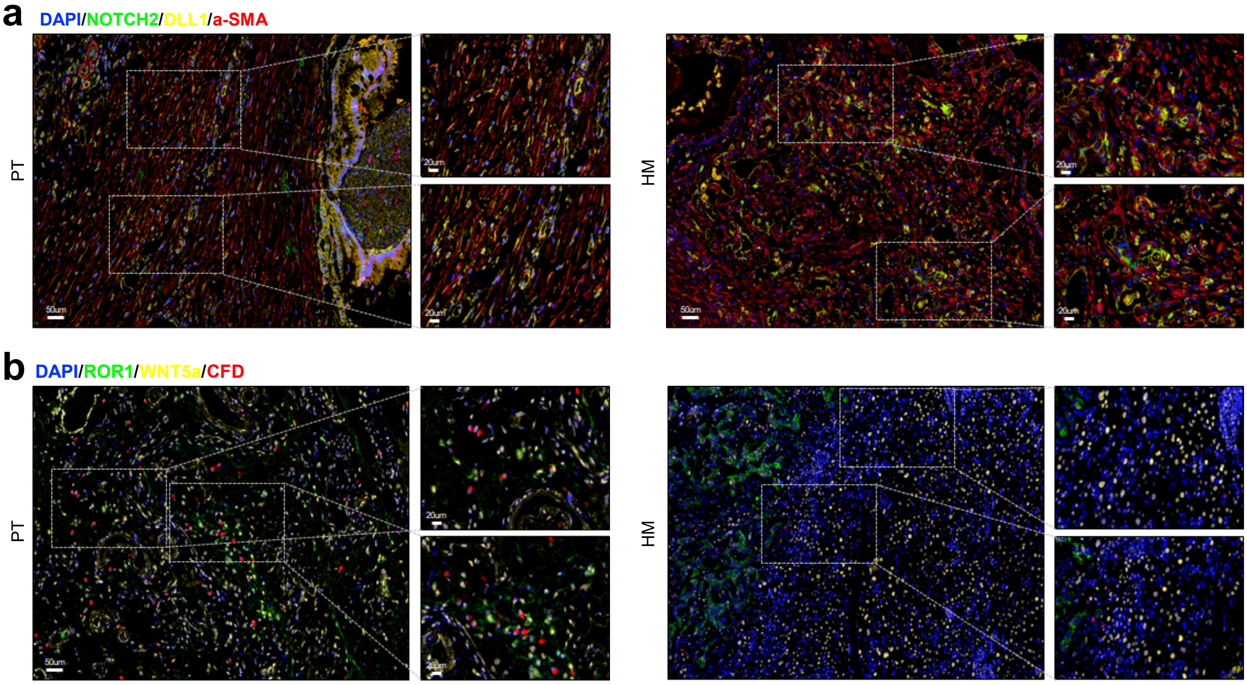

Supplementary Fig. 14. Validate the interaction of two ligand-receptor pairs by using IF staining experiments. Related to Figure 7. **(a)** Immunofluorescent staining showing co-localization of DAPI (blue), NOTCH2(green), DLL1(yellow) and a-SMA(red) in PT and HM tissues (n = 3). Scale bars of each group, 50μm (left) and 20μm (right). **(b)** Immunofluorescent staining showing co-localization of DAPI(blue), ROR1(green), WNT5a(yellow) and CFD(red) in PT and HM tissues (n = 3). Scale bars of each group, 50μm (left) and 20μm (right).

Supplementary Tables

Supplementary Table 1. Clinical histopathological parameters of patients Clinical and pathology details for patients analysed by scRNA-Seq in this study.

Patient ID	Diagnosis	Stage	Liver Metastasis	Operation
P01	PDAC	IV	1	DP
P02	PDAC	IV	1	TP
P03	PDAC	IV	1	DP
P04	PDAC	IV	1	EUS-FNA

Supplementary References

[1] Peng, J. *et al.*, Single-cell RNA-seq highlights intra-tumoral heterogeneity and malignant progression in pancreatic ductal adenocarcinoma. *CELL RES* **29** 725 (2019).

[2] Yang, J. *et al.*, Integrated genomic and transcriptomic analysis reveals unique characteristics of hepatic metastases and pro-metastatic role of complement C1q in pancreatic ductal adenocarcinoma. *GENOME BIOL* **22** 4 (2021).
