# Supplementary material for: Single cell transcriptomic analyses implicate an immunosuppressive tumor microenvironment in pancreatic cancer liver metastasis
Source: Nat Commun. 2023 Aug 23;14:5123. doi: 10.1038/s41467-023-40727-7 (PMC10447466; doi:10.1038/s41467-023-40727-7)
Supplement: Supplementary file 2 — Description of Additional Supplementary Files [file 41467_2023_40727_MOESM2_ESM.pdf]

Description of Additional Supplementary Files

Supplementary Data 1
The differentially expressed genes across cell clusters in the integrated cell map. Relevant to Figure 1 and Supplementary Figure 1.

Supplementary Data 2
The differentially expressed genes across cell subtypes of ductal cells. Relevant to Figure 2 and Supplementary Figure 2.

Supplementary Data 3
The pathways enriched in cell states of ductal cells by GSEA analyses. Relevant to Supplementary Figure 5.

Supplementary Data 4
The differentially expressed genes across cell subtypes of stromal cells (including fibroblasts, endothelial cells, endocrine cells and acinar cells). Relevant to Supplementary Figure 7.

Supplementary Data 5
The differentially expressed genes across cell subtypes of myeloid cells. Relevant to Figure 5 and Supplementary Figure 10.

Supplementary Data 6
The pathways enriched in cell subtypes of myeloid cells by GSEA analyses. Relevant to Supplementary Figure 10.

Supplementary Data 7
The differentially expressed genes across cell subtypes of lymphoid cells. Relevant to Figure 6.
