# Supplementary material for: Single cell transcriptomic analyses implicate an immunosuppressive tumor microenvironment in pancreatic cancer liver metastasis
Source: Nat Commun. 2023 Aug 23;14:5123. doi: 10.1038/s41467-023-40727-7 (PMC10447466; doi:10.1038/s41467-023-40727-7)
Supplement: Supplementary file 10 — Reporting Summary [file 41467_2023_40727_MOESM10_ESM.pdf]

nature portfolio

NCOMMS-22-08833B
Corresponding author(s): Ying Lv, Dijun Chen, Xiaoping Zou, Chao Yan

Last updated by author(s): Apr 22, 2023

Reporting Summary

Nature Portfolio wishes to improve the reproducibility of the work that we publish. This form provides structure for consistency and transparency in reporting. For further information on Nature Portfolio policies, see our Editorial Policies and the Editorial Policy Checklist.

Statistics

For all statistical analyses, confirm that the following items are present in the figure legend, table legend, main text, or Methods section.

n/a	Confirmed	
☐	☒	The exact sample size (*n*) for each experimental group/condition, given as a discrete number and unit of measurement
☐	☒	A statement on whether measurements were taken from distinct samples or whether the same sample was measured repeatedly
☐	☒	The statistical test(s) used AND whether they are one- or two-sided<br>*Only common tests should be described solely by name; describe more complex techniques in the Methods section.*
☒	☐	A description of all covariates tested
☒	☐	A description of any assumptions or corrections, such as tests of normality and adjustment for multiple comparisons
☐	☒	A full description of the statistical parameters including central tendency (e.g. means) or other basic estimates (e.g. regression coefficient) AND variation (e.g. standard deviation) or associated estimates of uncertainty (e.g. confidence intervals)
☐	☒	For null hypothesis testing, the test statistic (e.g. *F*, *t*, *r*) with confidence intervals, effect sizes, degrees of freedom and *P* value noted<br>*Give P values as exact values whenever suitable.*
☒	☐	For Bayesian analysis, information on the choice of priors and Markov chain Monte Carlo settings
☒	☐	For hierarchical and complex designs, identification of the appropriate level for tests and full reporting of outcomes
☒	☐	Estimates of effect sizes (e.g. Cohen's *d*, Pearson's *r*), indicating how they were calculated

Our web collection on statistics for biologists contains articles on many of the points above.

Software and code

Policy information about availability of computer code

Data collection	No software was used for data collection.
Data analysis	Whole-exome sequencing<br>Whole exome capture was performed using an Agilent SureSelect Human All Exon V6 kit. Exome sequencing data were aligned to the GRCh38 human reference genome with BWA (version 0.7.15)53. After sorting aligned reads using samtools (version 1.9), we used GATK (version 4.3.0.0) to mark and remove duplicate reads and perform base mass recalibration. The MuTect2 algorithm54 was used to identify somatic mutations. MuTect2 identifies candidate somatic mutations by Bayesian statistical analysis of bases and their qualities in the tumor and normal BAMs at a given genomic locus. Variants with mutation allele frequency (MAF) >0.7 were filtered. Allelic copy numbers in exome sequencing data were estimated using Sequenza with the default options.<br><br>10x single-cell sequencing<br>Cell suspension was loaded to the Chromium Single-Cell v2 3′ and 5′ Chemistry Library, catching 5000 to 10000 cells position. Library construction was sequenced on the NextSeq 500 platform (Illumina), receiving 26, 8 and 98 cycles run for Read 1, i7 index and Read 2. All steps were performed according to the manufacturer’s standard protocol.<br><br>scRNA-seq data processing, cluster annotation and data integration<br>The 10x Chromium single-cell RNA sequencing (scRNA-seq) data were processed using CellRanger (v3.1.0; 10x Genomics) for alignment, barcode assignment and unique molecular identifier (UMI) counting (using the genome reference set GRCh38-3.0.0). Filtered count matrices were converted to sparse matrices using the Seurat package (v3.2.3)55, and cells expressing less than 200 genes as well as with more than 20% mitochondrial reads, were excluded from the downstream analysis. The ‘doubletFinder_v3’ method from the DoubletFinder package (v2.0.3)56 was applied for additional cell filtering. Filtered data were then log normalized and scaled, with cell-cell variation due to UMI counts

and percent mitochondrial reads regressed out.
To avoid batch effects among samples and experiments, integration of single-cell data was performed using Seurat's canonical correlation analysis (CCA) integration method. A total of 2000 features for anchoring (the 'FindIntegrationAnchors' function) and 30 dimensions for alignment ('IntegrateData') were used. Cell clustering was performed by 'FindClusters' function at a resolution of 0.8 and the top 20 genes were used to define cell identity. Dimensionality reduction was performed with 'RunUMAP' function and visualized by Uniform Manifold Approximation and Projection (UMAP). For subgroup cell clustering, cells of different types were extracted separately and clustered by their respective first 30 principal components (PCs) using different resolutions based on visual inspection.

Identification of signature genes
We applied the 'FindAllMarkers' function in Seurat to identify specific genes for each cell subset. For the selection of marker genes specific to each cell cluster/subset, we calculated the log2 fold change (log2FC) between two groups (a cell cluster/subset vs. other cells) using the 'FindMarkers' function with the Wilcoxon rank-sum test (default parameters).

CNV estimation in ductal cells
The InferCNV package (version 1.6.0)57 was used to infer CNVs in EPCAM+ ductal cells and to recognize cancer cells with default parameters. The CNV signal for individual cells was estimated with a 100-gene sliding window. Genes with a mean count of less than 0.1 across all cells were filtered out before analysis and the signal were denoised using a dynamic threshold of 1.3 s.d. from the mean. Ductal cells were divided into four groups based on CNV accumulation scores: normal, low, medium, high in the analysis.

Gene signatures
Inflammation-associated genes, including IFNG, IFNGR1, IFNGR2, IL10, IL12A, IL12B, IL12RB1, IL12RB2, IL13, IL17A, IL17F, IL18, IL18R1, IL18RAP, IL1A, IL1B, IL2, IL21, IL21R, IL22, IL23A, IL23R, IL2RG, IL4, IL4R, IL5, IL6, JUN, NFKB1, RELA, RORA, RORC, S100A8, S100A9, STAT1, STAT3, STAT4, STAT6, TGFB1, TGFB2, TGFB3, and TNF, were obtained from previous publication by Smillie et al.58.

The cytotoxic gene list consists of 12 genes that translate to effector cytotoxic proteins (GZMA, GZMB, GZMH, GZMK, GZMM, GNLY, PRF1, and FASLG) and well-described cytotoxic T cell activation markers (IFNG, TNF, IL2R, and IL2). The list of genes used for dysfunctional T cells were obtained from Li et al.59 and the TAM gene list from Cassetta et al.60. Clinically targetable receptor or ligand immune modulator markers expressed on the surface of cells were taken from Wu et al.28.

Pathway analysis
Differentially expressed genes (DEGs) were detected by the 'FindAllMarkers' function in Seurat, using |FC| > 2 and adjusted p-value < 0.05 as the cut-off values. GO enrichment analysis on DEGs in this study were performed by the clusterProfiler package61. GSVA was conducted using the GSVA package62. Differences between different cell groups were calculated by the 'FindMarkers' function in the Seurat package.

Pseudotime analysis by Monocle
Ductal cell and CAF cell developmental trajectories were inferred using Monocle2 (version 2.99.3)63 with default parameters as recommended by the developers. Firstly, integrated gene expression matrices from specific cell type were exported from Seurat into Monocle to construct a CellDataSet. Secondly, the 'setOrderingFilter' function was applied to sort cells with the variable genes identified by the function of 'differentialGeneTest' (cutoff of q<0.001). Finally, after dimensionality reduction using the 'reduceDimension' function (using the 'DDRTree' reduction method), a series of representative key role genes were revealed along the differentiation progress by the 'plot_pseudotime_heatmap' function. Dimensionality reduction was performed with no normalization and the 'DDRTree' reduction method in the 'reduceDimension' step. The visualization function "plot_cell_trajectory" were used to plot each group along the same pseudotime trajectory.

RNA velocity
We used scVelo64 (version 0.2.3) to calculate the single-cell trajectory/directionality using spliced and unspliced reads from the pre-aligned bam files. From loom files produced by the command-line tool, we subset the exact same cells that were previously selected for Monocle trajectory analysis. RNA velocity, latent time, root, and terminal states were calculated using the dynamical velocity model.

Cell-cell interaction analysis
Cell-cell interactions among the cell types were estimated by CellPhoneDB (v2.1.1)34 with default parameters (20% of cells expressing the ligand/receptor) and using the version 2.0.0 of the database. CellPhoneDB infers the potential interaction strength between two cell subsets based on the gene expression level of a receptor-receptor pair. The significance of interaction is assessed through a permutation test (1000 times). The normalized gene expression was used as input. Interactions with p-value < 0.05 were considered significant. We only considered ligand-receptor interactions based on the annotation from the database, for which only and at least one partner of the interacting pair was a receptor, thus discarding receptor-receptor interactions and other interactions not involving a receptor.

Correlation to public datasets
Using the Cancer Genome Atlas (TCGA) PDAC bulk RNA-seq datasets, the relative abundance of subtypes of ductal cells was predicted by CIBERSORTx21 algorithm (https://cibersortx.stanford.edu/) with default parameters. The tumor samples were divided into two groups based on the estimated relative cell abundance. Overall survival analysis was performed with Cox proportional hazards regression using 'coxph' from the R package Survival. Kaplan-Meier plots were used to assess the prognostic value of cell types and to explore their effect in PDAC cancer progression.

Survival analysis
RNA-seq and clinical data of PDAC samples were obtained from TCGA to evaluate the prognostic effects of gene sets derived from specific cell states. To assess the impact of specific differentially expressed marker genes on PDAC cancer progression, the tumor samples were divided into two groups with high 50% and low 50% of the mean expression of the target genes. Survival curves were performed by the Kaplan-Meier method with the Survival package v.2.44, and visualized using the 'ggsurvplot' function of the survminer package. Significance was assessed by the log-rank test statistics (p-values) between two groups.

For manuscripts utilizing custom algorithms or software that are central to the research but not yet described in published literature, software must be made available to editors and reviewers. We strongly encourage code deposition in a community repository (e.g. GitHub). See the Nature Portfolio guidelines for submitting code & software for further information.

Data

Policy information about availability of data

All manuscripts must include a data availability statement. This statement should provide the following information, where applicable:

- Accession codes, unique identifiers, or web links for publicly available datasets
- A description of any restrictions on data availability
- For clinical datasets or third party data, please ensure that the statement adheres to our policy

Processed scRNA-seq data from this study are also available through the Gene Expression Omnibus under accession number GSE197177. The raw sequence data reported in this paper have been deposited in the Genome Sequence Archive (GSA-Human: HRA004556 (scRNA-seq); GSA-Human: HRA004625 (WES)) that are publicly accessible at https://ngdc.cncb.ac.cn/search/?dbId=hra&q=HRA004556 (scRNA-seq) and https://ngdc.cncb.ac.cn/search/?dbId=hra&q=HRA004625 (WES). For public datasets analysis, Peng et al.'s dataset5 (including 11 NT and 14 PT samples) was retrieved from the Genome Sequence Archive under the accession number GSA: CRA001160. And Yang et al.' dataset12 (including 6 NT, 13 PT and 14 HM samples) was retrieved from GSE151580. The inflammation-associated genes (Fig. 1b) were obtained from previous publication by Smillie et al.58, whose data were deposited in Single Cell Portal: SCP259. The TCGA PAAD dataset was obtained from GDC data portal (https://portal.gdc.cancer.gov/projects/TCGA-PAAD). Source data are provided with the paper.

Research involving human participants, their data, or biological material

Policy information about studies with human participants or human data. See also policy information about sex, gender (identity/presentation), and sexual orientation and race, ethnicity and racism.

Reporting on sex and gender	The patients included males and females, aged 49-73, as the information on sex and gender was not relevant in our study.
Reporting on race, ethnicity, or other socially relevant groupings	N/A
Population characteristics	Four patients who were pathologically diagnosed with PDAC with liver metastases were enrolled into this study. None of the patients received any antitumor therapy prior to surgery. Half of the patients were male, and above 60 years old at the time of diagnosis.
Recruitment	Patients who fit the clinical criteria and consented to the study were selected for further study. Only those patients who have synchronously resected pancreatic and liver tissue samples were enrolled for further analysis. There are no self selection bias.
Ethics oversight	The study protocol was reviewed and approved by the Ethics Committee of the First Affiliated Hospital of Soochow University and Nanjing Drum Tower Hospital. All patients provided written informed consent for sample collection and data analyses prior to operation.

Note that full information on the approval of the study protocol must also be provided in the manuscript.

Field-specific reporting

Please select the one below that is the best fit for your research. If you are not sure, read the appropriate sections before making your selection.

☒ Life sciences ☐ Behavioural & social sciences ☐ Ecological, evolutionary & environmental sciences

For a reference copy of the document with all sections, see nature.com/documents/nr-reporting-summary-flat.pdf

Life sciences study design

All studies must disclose on these points even when the disclosure is negative.

Sample size	Eight fresh tissues from four PDAC patients (P1-P4) were enrolled in this study, including three surgically resected primary pancreatic tumors (PT) and paired hepatic metastases (HM), as well as one normal pancreatic tissue (NT) from one of the three patients (1 NT-PT-HM trio and 2 PT-HM pairs). Besides, one hepatic metastasis biopsy obtained by endoscopic ultrasound-guided fine needle aspiration (EUS-FNA) from a fourth patient was also included. The sample size in this study (n=4 patients) is limited by the availability of surgically resected tumor tissues.
Data exclusions	Filtered count matrices were converted to sparse matrices using the Seurat package (v3.2.3), and cells expressing less than 200 genes as well as with more than 20% mitochondrial reads, were excluded from the downstream analysis. Cells expressing less than 200 genes and with more than 20% mitochondrial reads are considered as apoptotic or "dead" cells, which are inappropriate for further analysis.
Replication	This is not applicable since no technical replicates were used, only biological replicates. Indeed, due to very high costs of scRNA-seq and the inaccessibility to liver metastasis tissues in most PDAC patients, we could not include technical replicates for the single-cell analyses.
Randomization	Patients were not randomized in this study because randomization is not relevant to our study design. The samples were assigned to groups based on their tissue origin (pancreas or liver).
Blinding	There was no blinding in this study. The analyses in this study do not involve clinical research.

Reporting for specific materials, systems and methods

We require information from authors about some types of materials, experimental systems and methods used in many studies. Here, indicate whether each material, system or method listed is relevant to your study. If you are not sure if a list item applies to your research, read the appropriate section before selecting a response.

Materials & experimental systems

n/a	Involved in the study
☐	☒ Antibodies
☒	☐ Eukaryotic cell lines
☒	☐ Palaeontology and archaeology
☒	☐ Animals and other organisms
☒	☐ Clinical data
☒	☐ Dual use research of concern
☒	☐ Plants

Methods

n/a	Involved in the study
☒	☐ ChIP-seq
☒	☐ Flow cytometry
☒	☐ MRI-based neuroimaging

Antibodies

Antibodies used

The antibodies used in multiplexed immunofluorescence staining include RGS5 (rabbit, 1:300, 11590-1-AP, Proteintech), ACTA2/ smooth muscle actin (rabbit, 1:500, 14395-1-AP, Proteintech), Pan Cytokeratin (mouse, 1:500, 53-9003-82, Invitrogen), FN1 (mouse, 1:300, 66042-1-Ig, Proteintech) , S100A8 (rabbit, 1:200, 15792-1-AP, Proteintech) , MPO (rabbit, 1:1000, ab208670, Abcam), CD4 (mouse, 1:500, 67786-1-Ig, Proteintech), CD8 (mouse, 1:4000, 66868-1-Ig, Proteintech), FOXP3 (mouse, 1:500, ab20034, Abcam) , GNLY/Granulysin (rabbit, 1:4000, ab241333, Abcam) ▯CD68 (rabbit, 1:400,76437, Cell Signaling) and CCL18 (rabbit, 1:200, 22303-1-AP, Proteintech). LITAF (rabbit, 1:200, 16797-1-AP, Proteintech) was used in immunohistochemistry staining. The secondary antibodies used in this study included Goat Anti-Rabbit IgG H&L HRP (1:2000, ab205718, Abcam) and the supplied antibodies from the PANO 5-plex IHC kit (Cat# 10002100100, Panovue, Beijing, China).

Validation

All antibodies in this study were commercially purchased and have been validated by the vendors for species and application. Validation data are available from the respective vendor's respective websites.
